# Research progress on the role of lncRNA–miRNA networks in regulating adipogenic and osteogenic differentiation of bone marrow mesenchymal stem cells in osteoporosis

**DOI:** 10.3389/fendo.2023.1210627

**Published:** 2023-08-14

**Authors:** Fangyu An, Xiaxia Wang, Chunmei Wang, Ying Liu, Bai Sun, Jie Zhang, Peng Gao, Chunlu Yan

**Affiliations:** ^1^ Teaching Experiment Training Center, Gansu University of Chinese Medicine, Lanzhou, Gansu, China; ^2^ School of Tradional Chinese and Western Medicine, Gansu University of Chinese Medicine, Lanzhou, Gansu, China; ^3^ School of Basic Medicine, Gansu University of Chinese Medicine, Lanzhou, Gansu, China

**Keywords:** osteoporosis, bone marrow mesenchymal stem cells, adipogenic-osteogenic differentiation, lncRNA-miRNA network, crosstalk regulation

## Abstract

Osteoporosis (OP) is characterized by a decrease in osteoblasts and an increase in adipocytes in the bone marrow compartment, alongside abnormal bone/fat differentiation, which ultimately results in imbalanced bone homeostasis. Bone marrow mesenchymal stem cells (BMSCs) can differentiate into osteoblasts and adipocytes to maintain bone homeostasis. Several studies have shown that lncRNAs are competitive endogenous RNAs that form a lncRNA–miRNA network by targeting miRNA for the regulation of bone/fat differentiation in BMSCs; this mechanism is closely related to the corresponding treatment of OP and is important in the development of novel OP-targeted therapies. However, by reviewing the current literature, it became clear that there are limited summaries discussing the effects of the lncRNA–miRNA network on osteogenic/adipogenic differentiation in BMSCs. Therefore, this article provides a review of the current literature to explore the impact of the lncRNA–miRNA network on the osteogenic/adipogenic differentiation of BMSCs, with the aim of providing a new theoretical basis for the treatment of OP.

## 1 Introduction

Osteoporosis (OP) is the most common systemic metabolic bone disease among postmenopausal (1), middle-aged, and elderly women and is characterised by an imbalance in bone remodelling and formation that results in decreasing bone strength and increasing fracture risk. Overall, OP is a major cause of physical disability and death among middle-aged and elderly individuals (2). With an increase in the aging population worldwide, the global prevalence of OP increases annually (3). Over 200 million people worldwide have been diagnosed with OP (4), making this condition a serious public health issue that negatively impacts people’s health and imparts a corresponding economic burden (3).

Increasing evidence suggests (5–8) that bone marrow mesenchymal stem cells (BMSCs) can differentiate into multiple cell types and self-renew; therefore, these cells may play an important role in bone homeostasis and regeneration. Abnormalities in BMSC differentiation are closely associated with the occurrence of OP (9). Therefore, understanding the differentiation mechanism of BMSCs can assist in the identification of effective treatment methods for OP.

BMSCs are adult stem cells that are derived from the mesoderm and have the potential to self-renew and differentiate into osteoblasts (10–13), adipocytes, and other multi-directional cells. Therefore, differentiation of BMSCs into osteoblasts, rather than adipocytes, could potentially assist OP treatment. However, during aging, or other pathological stimuli, BMSCs preferentially differentiate into adipocytes, resulting in an increase in bone marrow adipocytes and a decrease in osteoblasts and bone formation (14, 15). As a metabolic bone disease, OP is characterised by a decrease in osteoblasts and accumulation of bone marrow adipocytes in the bone marrow compartments (16). Therefore, establishing a method to regulate the balance of osteogenic/adipogenic differentiation in BMSCs to restore bone homeostasis and reduce bone resorption is of great significance for bone health and OP treatment.

Osteogenic and adipogenic differentiation of BMSCs is controlled by regulator gene expression, transcriptional, and post-transcriptional mechanisms (17). Numerous non-coding RNA (ncRNAs) are involved in post-transcriptional regulation (18). In recent years, the key regulatory role of ncRNAs in skeletal system diseases has gained increasing recognition; therefore, ncRNAs are considered novel targets for the treatment of such diseases (19). Several studies have confirmed that long chain non-coding RNA (lncRNAs) and microRNAs (miRNAs) are important regulators of bone development and homeostasis (20–23); therefore, these ncRNAs have been identified as important regulators of BMSC differentiation. LncRNA is considered a natural sponge of miRNA, specifically binding to miRNA via competitive mechanisms, thereby forming a lncRNA–miRNA regulatory network; this mechanism plays an important regulatory role in the osteogenic adipogenic differentiation of BMSCs and is, therefore, expected to become a future target for OP treatment (24–27).

## 2 The lncRNA–miRNA network in osteoporosis

LncRNAs are transcripts that are >200 nucleotides long and lack protein coding domains; Therefore, these RNAs do not have, or possess very limited, translational ability (28). Alternatively, lncRNAs regulate the expression of genes and proteins at the transcriptional (29), post-transcriptional, and epigenetic levels; additionally, they play a key role in the regulation of various biological processes, including cell differentiation, proliferation, and apoptosis (30). lncRNAs, one of the largest and most significantly diverse RNA families, have emerged in recent years as an interesting field of research (31). Prior studies have determined that lncRNA dysregulation is associated with the occurrence of many diseases; therefore, lncRNAs have been widely studied in the diagnosis and treatment of these diseases (32). In recent years, many studies have shown that lncRNAs play crucial roles in bone development via the regulation of osteogenic and adipogenic markers or key regulatory factors (33); these lncRNAs have been particularly implicated in the regulation of BMSC differentiation into osteogenic and adipogenic cells (34). In addition, lncRNAs can be used as competitive endogenous RNA (ceRNAs) to regulate the expression of genes and proteins by binding to miRNAs; therefore, lncRNAs can be used to regulate the osteogenic/adipogenic differentiation of BMSCs and may be important in the development of OP treatment (17) (35).

MiRNAs are small (18–25 nucleotides) endogenous non-coding single-stranded RNAs (36); by targeting the 3′-untranslated region (3′-UTR) of the target gene, miRNAs play a central role in the post-transcriptional regulation of protein coding genes, thereby participating in various important biological functions, such as cell differentiation, metabolism, proliferation, and apoptosis (37–39). Recent studies have found that miRNAs not only play a critical role in the proliferation and differentiation of mesenchymal stem cells, but also in the metabolic activities of bone cells (40–42). Specifically, miRNAs are an important molecular regulatory factor in the processes of bone remodelling and bone cell growth, differentiation, and function. By regulating BMSC differentiation, miRNAs maintain metabolic homeostasis within the bone, which ultimately affects bone metabolic homeostasis and formation. Therefore, miRNAs participate in the occurrence and development of OP and other bone diseases (20) (43, 44). A recent study identified a novel ceRNA that is necessary for the regulation of miRNAs and target genes. CeRNAs participate in the prevention and treatment of OP by regulating the expression of miRNAs and their downstream target genes (45, 46).

Recent studies have established that ceRNAs represent a novel mechanism of RNA-to-RNA interactions. These ceRNAs compete with mRNA to bind to miRNAs, thereby regulating the expression of downstream target genes and affecting physiological and pathological processes throughout the body (47, 48). Many lncRNAs act as ceRNAs by competing to bind to downstream miRNAs to suppress miRNA expression; therefore, these lncRNAs can disrupt the balance between miRNA and target gene expression, which may contribute to the pathogenesis of various human diseases (49, 50). LncRNAs regulate osteoblast proliferation and function via the corresponding ceRNA mechanism; therefore, abnormalities in this lncRNA–miRNA ceRNA network can contribute to the development of OP (34). Consequently, regulation of the lncRNA–miRNA network is expected to become a major topic within the field of OP prevention and treatment.

## 3 The lncRNA–miRNA network regulates osteogenic differentiation of BMSCs

BMSCs are the primary site of osteogenic differentiation and are, therefore, important research factors for the field of bone repair and OP treatment (51, 52). Increasing evidence suggests that lncRNA and miRNA not only individually regulate various transcription factors related to osteoblast differentiation, but also form a lncRNA–miRNA network via the corresponding ceRNA mechanism; this mechanism involves miRNA binding, resulting in the regulation of key transcription factors for osteogenesis via various pathways or by directly targeting osteoblast proliferation and differentiation (53–55). Overall, lncRNAs possess a crucial role in the osteogenic differentiation of BMSCs and have, consequently, become a popular research topic.

### 3.1 LncRNA–miRNA network regulation promotes osteogenic differentiation of BMSCs

**Figure 1:**
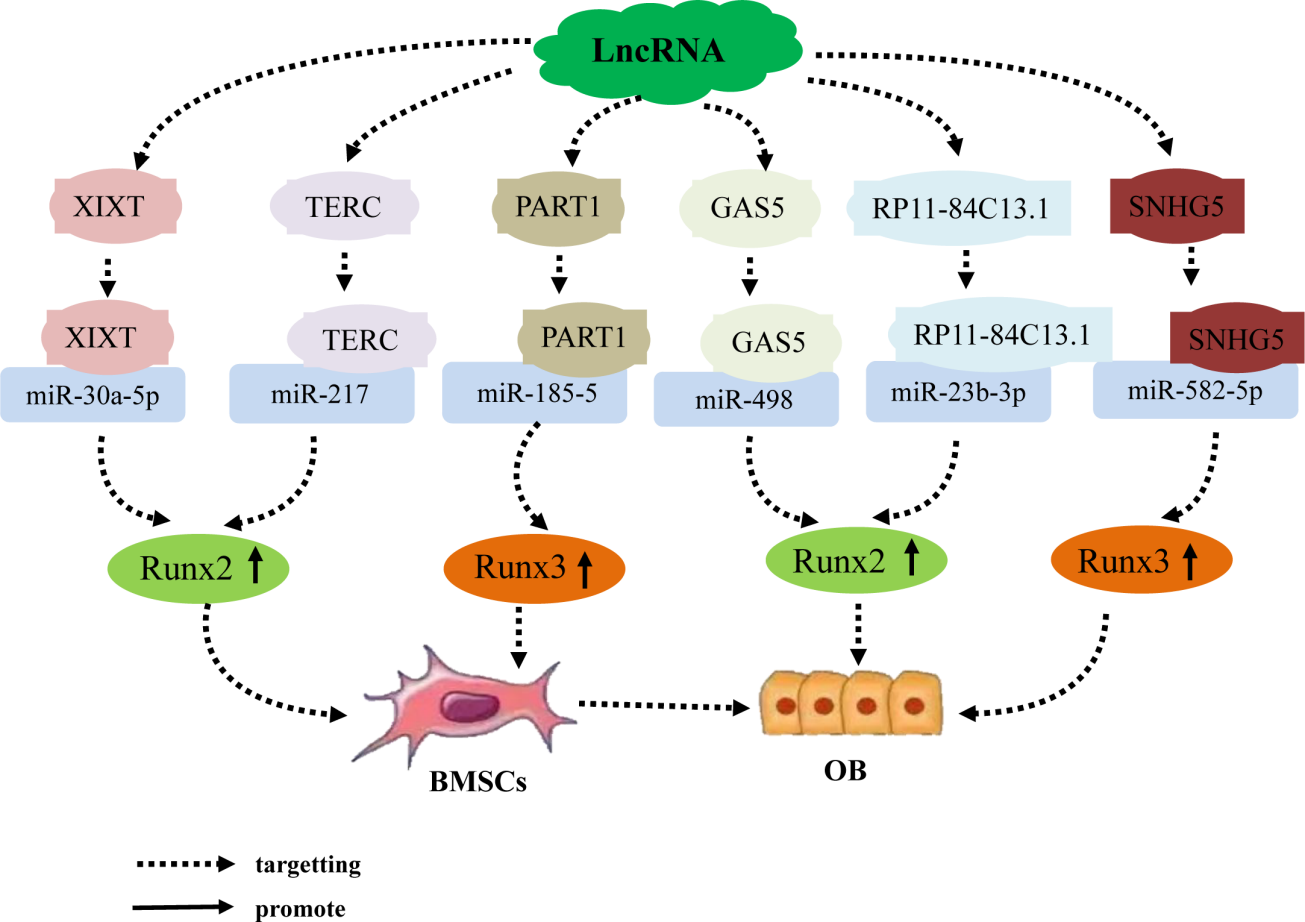
The lncRNA–miRNA network regulates the expression of Runx family members to promote osteogenic differentiation of BMSCs. Different lncRNAs bind to different miRNAs to inhibit miRNA expression, upregulate the expression of osteogenesis-related genes Runx2 and Runx3, and induce osteogenic differentiation of hBMSCs. An individual lncRNA can upregulate the expression of the osteogenesis-related factor Runx2 by targeting different miRNAs, thereby promoting osteogenic differentiation of hBMSCs.

Previous studies have found that the lncRNA–miRNA network plays an important role in promoting the osteogenic differentiation of BMSCs by regulating osteogenic transcription factors. Runt domain transcription factor X (Runx) is a highly conserved family of transcription factors that are involved in organ development, cell metabolic proliferation, and stem cell differentiation (56). The Runx family comprises three members: Runx1, Runx2, and Runx3. Runx2 has been confirmed to be a transcription factor specifically involved in osteogenic differentiation; it can, therefore, serve as a marker of osteoblast differentiation. Runx2 regulates the transcription of numerous genes and induces differentiation of BMSCs into osteoblasts. This mechanism is a necessary and sufficient condition for the differentiation of BMSCs into the osteoblast lineage and is important in the development of the skeletal system and bone metabolism (57–60). Numerous studies have established that lncRNA upregulates BMSC osteogenic differentiation by acting as an miRNA sponge, which inhibits miRNA via competitive binding to miRNA; ultimately, this forms a lncRNA–miRNA network that upregulates Runx2 expression. Zhang (61) et al. determined that the lncRNA-XIXT upregulates the expression of Runx2 by competitive adsorption of miRNA-30a-5p, Runx2 is the downstream target gene of miRNA-30a-5p, thereby inducing the osteogenic differentiation of human (h)BMSCs and alleviating OP. Gao (62) et al. found that the lncRNA TERC upregulates Runx2 expression by binding to miRNA 217, Runx2 is the downstream target gene of miRNA 217, thereby accelerating the osteogenic differentiation of hMSCs and alleviating the progression of OP. In addition, the lncRNAs GAS5 and RP11 target miRNA-498 and miR-23b-3p, respectively, both of which upregulate Runx2 expression, Runx2 is the downstream target gene of miRNA-498 and miR-23b-3p, thereby promoting osteogenic differentiation of hMSCs and alleviating the development of OP (63, 64). Further, Runx3 not only regulates the occurrence of diseases such as cancer, but also participates in the process of bone development (65). Zhang (66) Zheng (67) et al. found that the lncRNAs PART1 and SNHG5 act as ceRNAs to upregulate Runx3 expression by targeting miR-185-5p and miRNA-582-55p, respectively, Runx3 is the downstream target gene of miR-185-5p and miRNA-582-55p, thereby promoting the osteogenic differentiation of hBMSCs. The results suggest that LncRNA induces osteogenic differentiation of hBMSCs by targeting and binding to miRNA, inhibiting miRNA expression, upregulating the expression of osteogenic related genes Runx2 and Runx3 (**Figure 1**).

**Figure 2:**
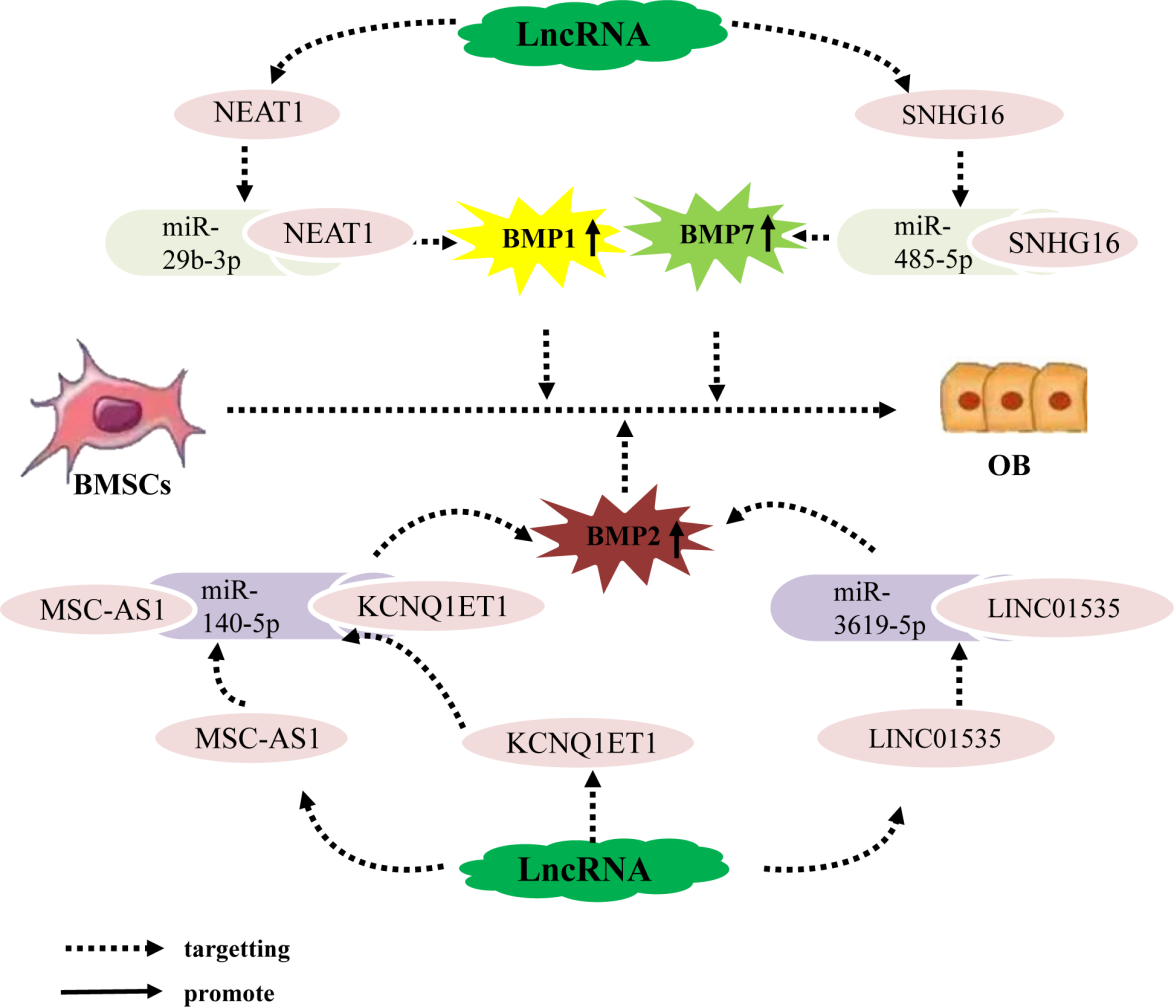
The lncRNA–miRNA network regulates the expression of BMP family members to promote osteogenic differentiation of BMSCs. Different lncRNAs bind to different miRNAs to inhibit miRNA expression, upregulate the expression of osteogenesis-related factors BMP1, BMP2, and BMP7, and promote the osteogenic differentiation of hBMSCs.

Bone morphogenetic protein (BMP) belongs to the TGF-β superfamily and is a multifunctional growth factor that is crucial for bone formation. BMP2 and BMP7 are key factors in osteogenic differentiation (68, 69); additionally, although BMP1 does not belong to TGF-β superfamily, unlike other BMPs, it can still induce skeletal development (70). Recent studies have shown that the lncRNA–miRNA network is an important regulator of BMP expression. For example, the lncRNA NEAT1 upregulates BMP1 expression by binding to miR-29b-3p, BMP1 is the downsteam target gene of miR-29b-3p, ultimately promoting the osteogenic differentiation of hBMSCs (71). Additionally, BMP2 is a well-established effective inducer of osteoblast differentiation in BMSCs. Zhang (72) Wang (73) Zhao (74) et al. found that the lncRNA MSC-AS1 binds to miRNA-140-5p, the lncRNA KCNQ1ET1 binds to miR-214, and the lncRNA LINC01535 competes to bind to miR-3619-5p,. All three of these lncRNAs inhibit the expression of miRNA-140-5p, miR-214, miR-3619-5p and upregulate BMP2, BMP2 is the downsteam target gene of miRNA-140-5p, miR-214, miR-3619-5p, thereby promoting the osteogenic differentiation of BMSCs and alleviating OP progression. In addition, BMP7 has been determined to regulate bone formation and enhance the osteoblast differentiation ability of BMSCs. For example, the lncRNA SNHG16 upregulates BMP7 expression by targeting miR-485-5p, BMP7 is the downsteam target gene of miR-485-5p, thereby promoting the osteogenic differentiation of hBMSCs (75). In conclusion, LncRNA promote osteogenic differentiation of hBMSCs by targeting and binding to miRNA, inhibiting miRNA expression, upregulating the expression of osteogenic related genes BMP1, BMP2, and BMP7 (**Figure 2**).

**Figure 3:**
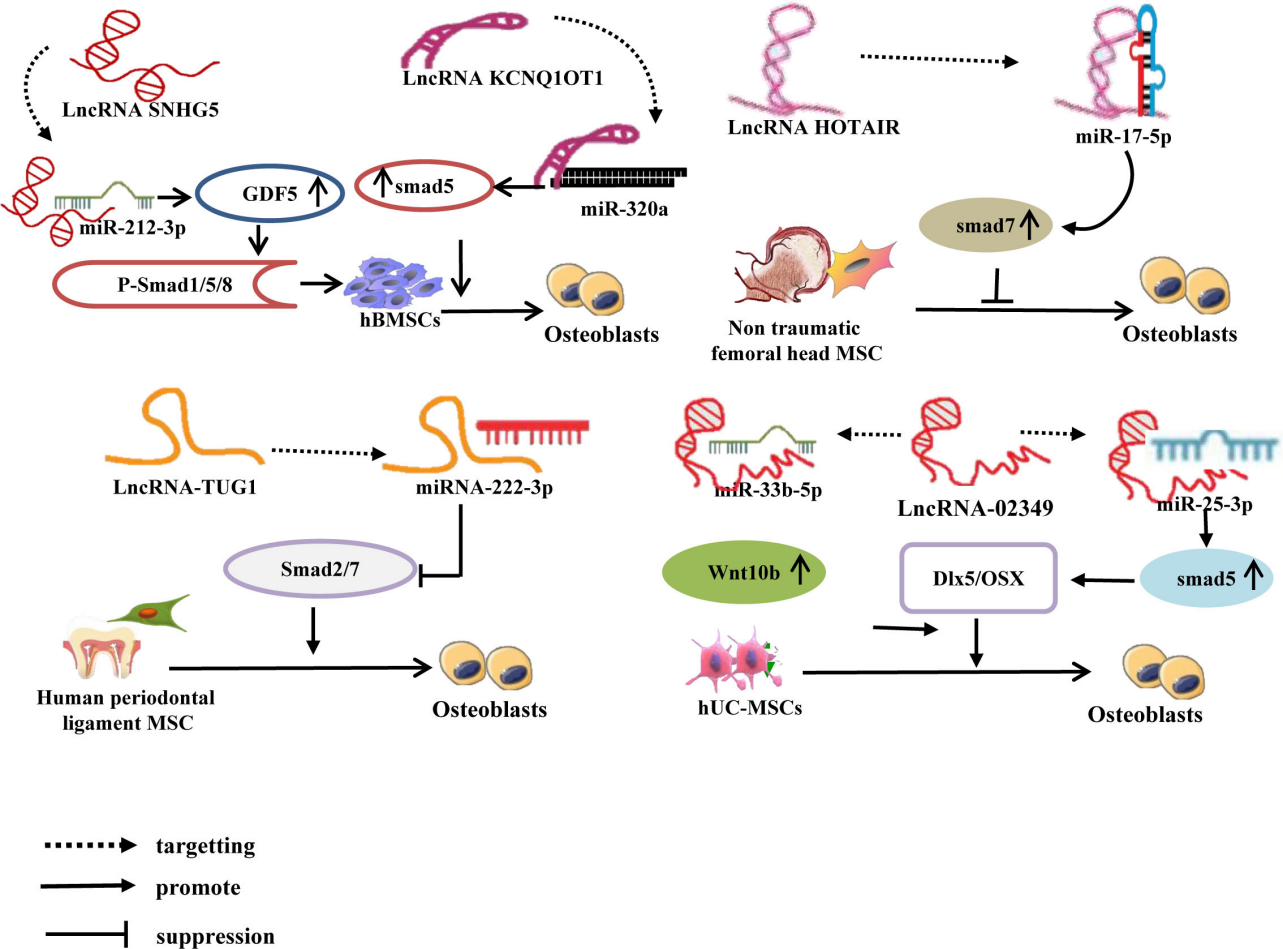
LncRNA-miRNA network regulates Smad to promote/inhibit MSC osteogenic differentiation. Different lncRNAs inhibit the expression of miRNAs by binding to different miRNAs, upregulate or inhibit the expression of downstream target genes of miRNAs, and induce osteogenic differentiation of MSCs.

Currently, three primary categories of Smad homologous proteins (SMAD)s family have been identified, specifically receptor-regulated Smad (R-Smad), common Smad (co-Smad), and inhibitory Smad (I-Smad) (76). Among them, R-SMADs consist of five members, namely 1, 2, 3, 5, and 8, which exhibit active involvement in specific signal transmission. Smad4 exclusively functions as a co-Smad, whereas I-Smad encompasses Smad6 and Smad7. Smad1, 5, and 8 are BMP receptors’ immediate downstream molecules. Smad2, 3, 4, and 7 are essential signaling molecules in the TGF-1/Smad signaling pathway, and they play a significant role in controlling bone metabolism balance in the BMP/Smad signaling pathway and the TGF-1/Smad signaling circuit, respectively (77–79). Recent research has discovered that the lncRNA-miRNA network controls the SMADs family and performs a regulatory function in mesenchymal stem cell (MSC) osteogenic development. For example, Han et al. (80) discovered that lncRNA SNHG5 controls GDF5 expression and stimulates Smad1/5/8 phosphorylation by sponge adsorption of miR-212-3p during hBMSC osteogenic differentiation, hence increasing hBMSC osteogenic differentiation. Moreover, during the osteogenic differentiation of hBMSCs, lncRNA KCNQ1OT1 adsorbs miR-320a via sponge, inhibiting the expression of miR-320a while increasing the expression of its downstream target gene Smad5, thereby promoting the osteogenic differentiation of hBMSCs (81). Other research (82) has found that during osteogenic differentiation of human umbilical cord mesenchymal stem cells (hUC-MSCs), lncRNA-02349 upregulates the expression of Smad5 and Wnt10b by sponge adsorption of miR-25-3p and miR-33b-5p, activating the Dlx5/OSX signaling pathway, and promoting osteogenic differentiation of hUC-MSCs. There have also been confirmed (83) that during the osteogenic differentiation of human periodontal ligament MSCs, lncRNA-TUG1 adsorbs miRNA-222-3p through sponge, limiting its downstream Smad2/7 expression and thereby favoring osteogenic differentiation of human periodontal ligament MSCs. Wei et al. discovered *in vitro* that lncRNA HOTAIR suppresses miR-17-5p expression by targeting miR-17-5p adsorption, upregulates its downstream target gene Smad7, and inhibits osteoblast development in non-traumatic femoral head MSCs (84). (**Figure 3**).

**Figure 4:**
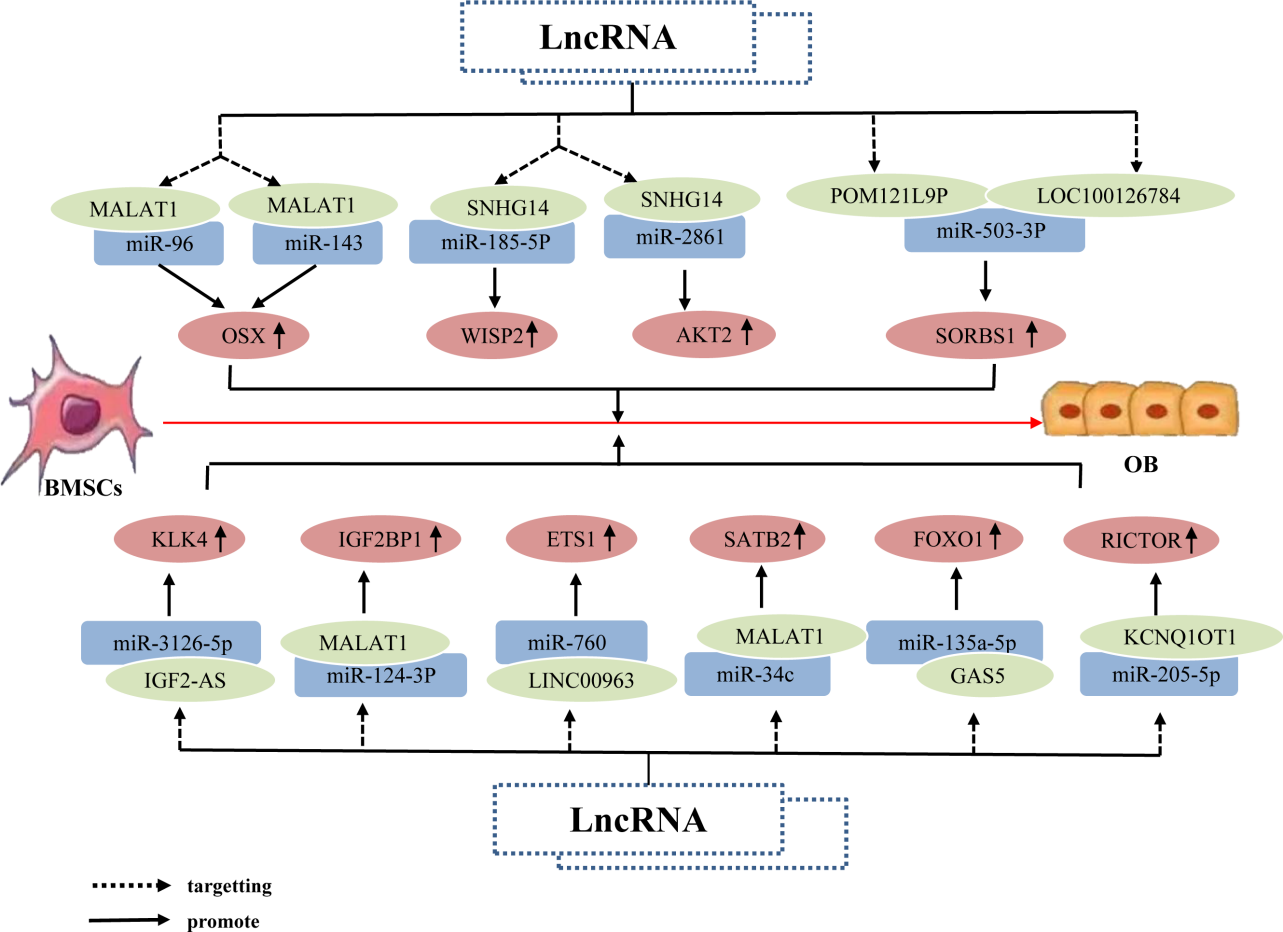
The lncRNA–miRNA network regulates osteogenesis-related factors to promote BMSC osteogenic differentiation. The same lncRNA can bind to multiple miRNAs to inhibit miRNA expression; this inhibition of different miRNAs can upregulate the same osteogenesis-related factor, Osx, alongside different osteogenesis-related factors WISP2 and AKT2, respectively, thereby promoting the osteogenic differentiation of hBMSCs. Different lncRNAs bind to different miRNAs; this inhibits corresponding miRNA expression, resulting in upregulation of the same osteogenesis-related factor SORS1, and promotion of osteogenic differentiation of hBMSCs. Different lncRNAs bind to different miRNAs and inhibit miRNA expression, thereby upregulating the expression of osteogenesis-related factors KLK4, IGF2BP1, SATB2, ETS1, RICTOR, and FOXO1, and ultimately promoting osteogenic differentiation of hBMSCs.

Research has shown that individual lncRNAs can play an important role in the osteogenic differentiation of BMSCs by targeting different miRNAs; through this inhibition of both miRNAs, osteogenesis-related factors downstream of these miRNAs can be upregulated. For example, the lncRNA MALAT1 binds to miR-96 and miR-143, upregulating Osx expression, thereby promoting the osteogenic differentiation of hBMSCs (85, 86). Some studies have also determined that individual lncRNAs can promote the osteogenic differentiation of BMSCs by targeting different miRNAs, which upregulate different osteogenesis-related factors downstream. For example, the lncRNA SNHG14 upregulates the expression of WISP2 and AKT2 by binding to miR-185-5p and miR-2861, respectively, thereby promoting osteogenic differentiation of hBMSCs (87, 88). Additionally, different lncRNAs can affect the osteogenic differentiation of BMSCs by both targeting the same miRNA, resulting in the upregulation of the same osteogenesis-related factor downstream of this miRNA. For example, the lncRNAs LOC100126784 and POM121L9P both bind to miR-503-5p, which reduces miR-503-5p expression, thereby upregulating SORS1 expression and promote the osteogenic differentiation of BMSCs (89). Other studies have also determined that the lncRNAs MALAT1, IGF2-AS, KCNQ1OT1, and GAS5 upregulate the expression of SATB2, KLK4, RICTOR, and FOXO1, respectively, by acting as miRNA sponges of miR-34c, miR-3126-5p, miR-205-5p, and miR-135a-5p, thereby enhancing osteoblastic activity and promoting the osteogenic differentiation of BMSCs (90–93). Further, the lncRNAs MALAT1 and LINC00963 act as ceRNAs, which competitively bind to miR-124-3p and miR-760 to upregulate the expression of IGF2BP1 and ETS1, respectively; ultimately, this promotes the osteogenic differentiation of BMSCs and inhibits OP progression (94, 95). Overall, the same LncRNA upregulates osteogenic related factors by binding to different miRNAs, different LncRNAs by binding to the same miRNA, and different LncRNAs by binding to different miRNAs, jointly promoting osteogenic differentiation of hBMSCs (**Figure 4**).

**Figure 5:**
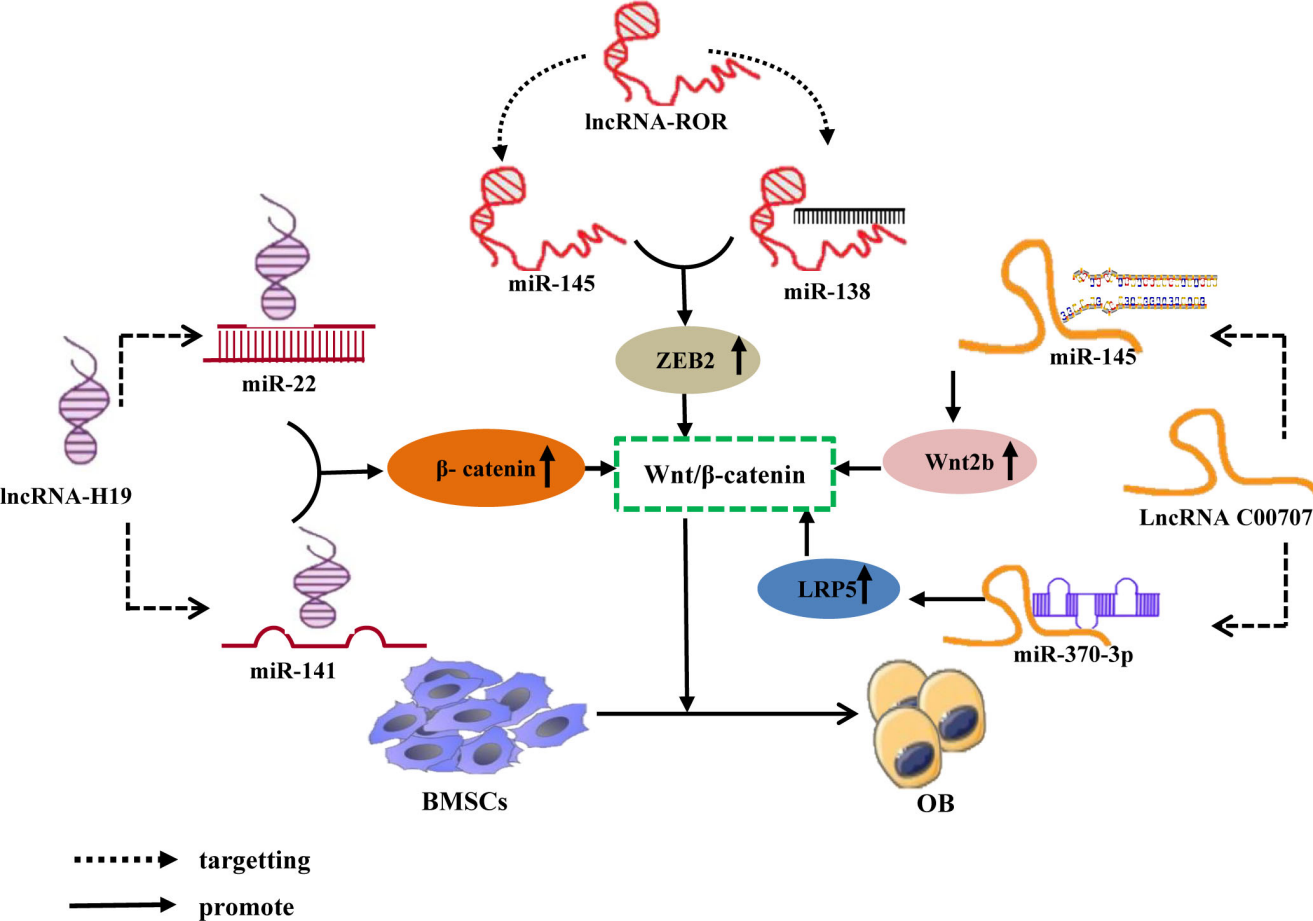
LncRNA-miRNA network regulates Wnt/β-Catenin signaling pathway promotes osteogenic differentiation of BMSCs. Different lncRNAs bind to different miRNAs to inhibit miRNA expression and upregulate the downstream target gene of miRNA, β-catenin, ZEB2, LRP5, and Wnt2b, All activate Wnt/β-The catenin signaling pathway promotes osteogenic differentiation of BMSCs.

Furthermore, research has confirmed that the lncRNA-miRNA network loop regulates BMSC osteogenic differentiation by interfering with osteogenic-related target genes, and its major mechanism may involve the regulation of osteogenic-related signaling pathways (96). As Wnt/β-catenin signaling pathway is a classic pathway for the differentiation of BMSCs (97), it consists primarily of transmembrane receptors on the Wnt family of extracellular proteins, cytoplasmic degradation complexes, and β-catenin (98). Recent studies have revealed that the lncRNA-miRNA network loop controls the Wnt/β-catenin signaling pathway and promotes the osteogenic differentiation of BMSCs. For example, Liang et al. (99) discovered that lncRNA-H19 absorbs miR-141 and miR-22 via sponges, inhibits their expression, and upregulates their common downstream target molecules β-catenin, thereby activating the Wnt/β-catenin pathway and promoting osteogenic differentiation of hMSCs *in vitro*. Some studies have also shown that lncRNA-ROR activates the Wnt/-catenin pathway, thereby promoting the osteogenic differentiation of human mesenchymal stem cells (hMSCs) by sponging miR-138 and miR-145, inhibiting their function, and elevating the expression of their common downstream target ZEB2 (100). Moreover, Cai (101) and Jia (102) et al. discovered via *in vitro* experiments that lncRNA C00707 competes to adsorb miR-145 and miR-370-3p, respectively, inhibiting their expression and upregulating the expression of downstream target proteins LRP5 and Wnt2b, thereby activating the Wnt/-catenin signaling pathway and promoting osteogenic differentiation of BMSCs. (**Figure 5**). And research has confirmed that the Wnt/-catenin signaling pathway can regulate the transcriptional expression of factors such as Runx2 and Osterix (103), Osterix is a downstream target gene of Runx2, and both play essential regulatory roles in osteoblast differentiation (104). And Wnt2b can regulate the gene and protein expression of Runx2 and promote the expression of Osterix. The aforementioned findings suggest that the lncRNA-miRNA network loop regulates the osteogenic differentiation of BMSC by regulating the expression of important genes, proteins, etc., which may be accomplished by interfering with osteogenic-related signaling pathways.

### 3.2 LncRNA–miRNA network regulation inhibits osteogenic differentiation of BMSCs

**Figure 6:**
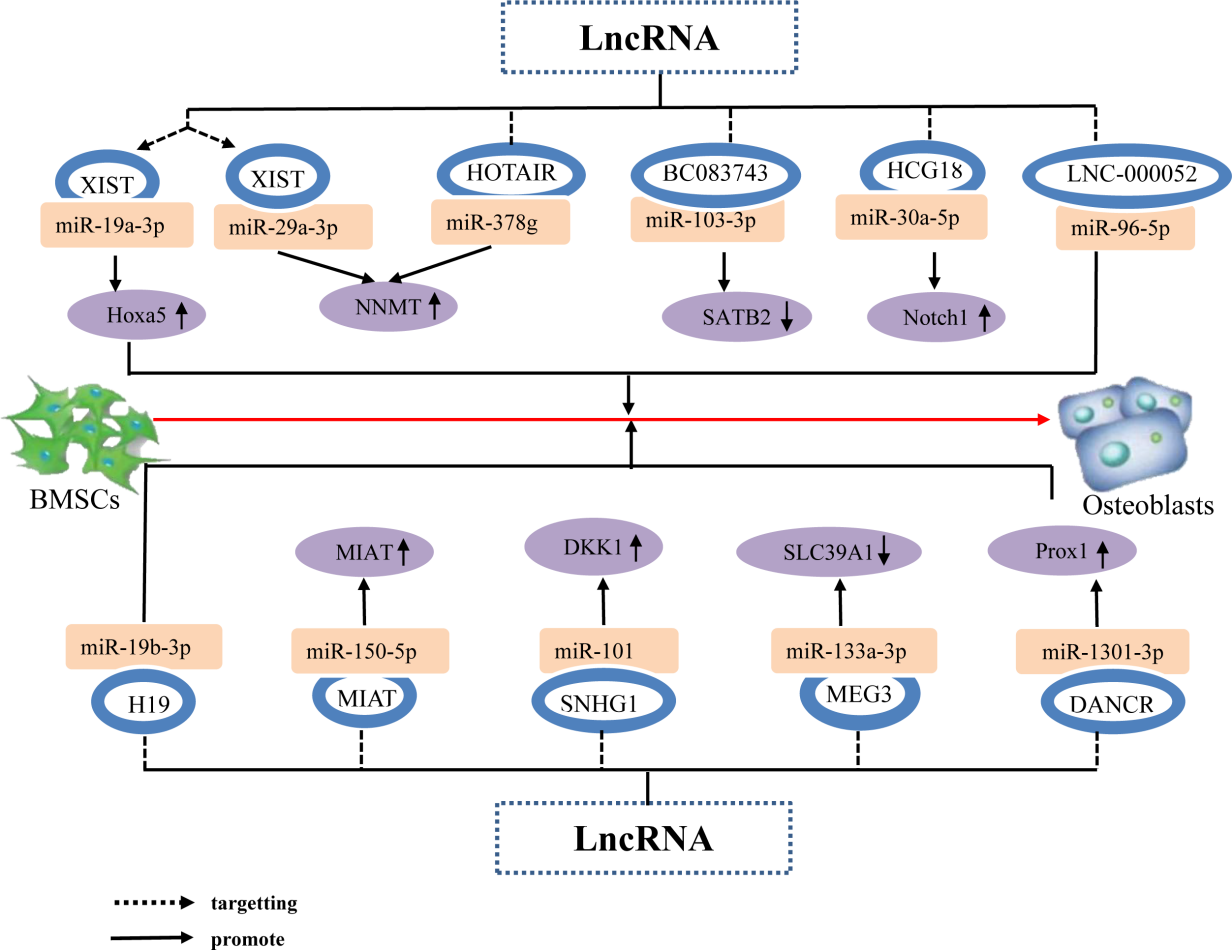
The lncRNA–miRNA network regulates osteogenic related factors and inhibits BMSC osteogenic differentiation. Individual lncRNAs bind to various miRNAs to inhibit miRNA expression, thereby upregulating the expression of osteogenesis-related factors Hoxa5 and NNMT, promoting the osteogenic differentiation of hBMSCs. Different lncRNAs bind to different miRNAs to inhibit miRNA expression, upregulate the expression of the same osteogenesis-related factor NNMT, and promote osteogenic differentiation of hBMSCs. Different lncRNAs bind to different miRNAs to inhibit or promote miRNA expression, resulting in direct or indirect stimulation of hBMSC osteogenic differentiation via the upregulation of the osteogenesis-related factors Notch1, MIAT, DKK1, and downregulation of SATB2 and SLC39A1.

Prior studies have determined that the lncRNA XIST enhances the expression of Hoxa5 and NNMT genes, which are downstream targets of miR-19a-3p and miR-29b-3p, respectively, by acting as a sponge for miR-19a-3p and miR-29b-3p, thereby inhibiting the osteogenic differentiation of BMSCs (105, 106). Additionally, Wang (107) Weng (108) Xiang (109) et al. determined that lncRNAs, namely HOTAIR, DANCR, and SNHG1, function as ceRNAs by sequestering miR-378g, miR-1301-3p, and miR-101, thereby repressing their expression and subsequently enhancing the expression of NNMT, PROX1, and DKK1, respectively. consequently, these lncRNAs inhibited the osteogenic differentiation of hBMSCs. Other studies have found that the lncRNAs HCG18 and MIAT upregulate the expression of Notch1 and MIAT by targeting miR-30a-5p and miR-150-5p, respectively, lncRNAs HCG18 and MIAT are the taget gene of miR-30a-5p and miR-150-5p, respectively, thereby inhibiting the osteogenic differentiation of BMSCs (110, 111). This indicates that individual and different lncRNAs can target different miRNAs to upregulate the expression of target genes downstream of the miRNAs and promote the osteogenic differentiation of BMSCs. Further, different lncRNAs can downregulate the expression of downstream target genes by targeting different miRNAs, thereby promoting osteogenic differentiation of BMSCs. For example, Lu (112) Wang (113) et al. determined that the lncRNA BC083743 downregulates SATB2 expression by targeting miR-103-3p, and lncRNA MEG3 downregulates SLC39A1 expression by promoting miR-133a-3p expression; overall, these jointly inhibiting the osteogenic differentiation of hBMSCs. In addition, lncRNA not only targets miRNA to form a lncRNA–miRNA network that regulates downstream miRNA molecules to promote hBMSC osteogenic differentiation, but can also directly target miRNA to promote hBMSC osteogenic differentiation. For example, the lncRNA LNC-00052 inhibits the expression of miR-96-5p and miR-19b-3p by binding to these miRNAs, both of which inhibit the osteogenic differentiation of BMSCs (114, 115). The results suggest that LncRNA Inhibition osteogenic differentiation of hBMSCs by targeting and binding to miRNA, inhibiting miRNA expression, up or down regulation the expression of osteogenic related factors (**Figure 6**).

These results indicate that in the process of BMSC osteogenic differentiation, the upregulation of individual or different lncRNAs can downregulate different miRNAs to inhibit their expression to promote the expression of downstream osteogenic-related factors, thereby promoting BMSC osteogenic differentiation. Downregulation of individual or different lncRNAs can inhibit or promote the expression of downstream osteogenesis-related factors by upregulating the expression of different miRNAs, thereby inhibiting the osteogenic differentiation of BMSCs. The results are shown in **Table 1**. Therefore, in the process of BMSC osteogenic differentiation, determining whether downregulation of different lncRNAs can promote or inhibit the expression of downstream adipogenesis-related factors by upregulating miRNA expression could be a potential direction for future research. Additionally, further research into other diseases is required to determine whether a single lncRNA or miRNA can upregulate various pathways to increase the expression of disease-associated molecules and stimulate disease progression.

**Table 1:** LncRNA miRNA network regulates osteogenic differentiation of bone marrow mesenchymal stem cells.

LncRNA	miRNA	Impact on miRNA	Impact on downstream target genes of miRNA	The relationship between target genes and networks	Molecular function	Refs
XIXT	30a-5p	Inhibition of 30a-5p expression	Runx2↑	downstream	Inducing osteogenic differentiation of hBMSCs	(61)
TERC	217	Inhibition of 217 expression	Runx2↑	downstream	Accelerate osteogenic differentiation of hMSCs	(62)
GAS5	498	Inhibition of 498 expression	Runx2↑	downstream	Promoting osteogenic differentiation of hBMSCs	(63)
RP11-84C13.1	23b-3p	Inhibition of 23b 3p expression	Runx2↑	downstream	Promoting osteogenic differentiation of hBMSCs	(64)
PART1	185-5	Inhibition of 185-5 expression	Runx3↑	downstream	Promoting osteogenic differentiation of hBMSCs	(66)
SNHG5	582-5p	Inhibition of 582-5p expression	Runx3↑	downstream	Promoting osteogenic differentiation of hBMSCs	(67)
NEAT1	29b-3p	Inhibition of 29b-3p expression	BMP1↑	downstream	Promoting osteogenic differentiation of BMSCs	(71)
MSC-AS1	140-5p	Inhibition of 140-5p expression	BMP2↑	downstream	Promoting osteogenic differentiation of BMSCs	(72)
KCNQ1ET1	214	Inhibition of 214 expression	BMP2↑	downstream	Promoting osteogenic differentiation of BMSCs	(73)
LINC01535	3619-5p	Inhibition of 3619-5p expression	BMP2↑	downstream	Promoting osteogenic differentiation of BMSCs	(74)
SNHG16	485-5p	Inhibition of 485-5p expression	BMP7↑	downstream	Promoting osteogenic differentiation of hBMSCs	(75)
SNHG5	212-3p	Inhibition of 212-3p expression	activation p-Smad1/5/8	downstream	Promoting osteogenic differentiation of BMSCs	(80)
CNQ1OT1	320a	Inhibition of 320a expression	Smad5↑	downstream	Promoting osteogenic differentiation of hBMSCs	(81)
02349	25-3p	Inhibition of 25-3p expression	Smad5↑	downstream	Promoting osteogenic differentiation of hUC-MSCs	(82)
TUG1	222-3p	Inhibition of 222-3p expression	Smad2/7↓	downstream	Promoting osteogenic differentiation of Human periodontal ligament MSC	(83)
HOTAIR	17-5p	Inhibition of 17-5p expression	Smad7↑	downstream	Inhibited osteogenic differentiation of non traumatic femoral head MSC	(84)
MALAT1	96	Inhibition of 96 expression	Osx↑	downstream	Promoting osteogenic differentiation of hBMSCs	(85)
MALAT1	143	Inhibition of 143 expression	Osx↑	downstream	Promoting osteogenic differentiation of hBMSCs	(86)
SNHG14	185-5p	Inhibition of 185-5p expression	WISP2↑	downstream	Promoting osteogenic differentiation of hBMSCs	(87)
SNHG14	2861	Inhibition of 2861 expression	AKT2↑	downstream	Promoting osteogenic differentiation of hBMSCs	(88)
LOC100126784	503-5p	Inhibition of 503-5p expression	SORBS1↑	downstream	Promoting osteogenic differentiation of BMSCs	(89)
POM121L9P	503-5p	Inhibition of 503-5p expression	SORBS1↑	downstream	Promoting osteogenic differentiation of BMSCs	(89)
MALAT1	34c	Inhibition of 34c expression	SATB2↑	downstream	Promoting osteogenic differentiation of BMSCs	(90)
IGF2-AS	3126-5p	Inhibition of 3126-5p expression	KLK4↑	downstream	Promoting osteogenic differentiation of BMSCs	(91)
KCNQ1OT1	205-5p	Inhibition of 205-5p expression	RICTOR↑	downstream	Promoting osteogenic differentiation of BMSCs	(92)
GAS5	135a-5p	Inhibition of 135a-5p expression	FOXO1↑	downstream	Promoting osteogenic differentiation of BMSCs	(93)
MALAT1	124-3p	Inhibition of 124-3p expression	IGF2BP1↑	downstream	Promoting osteogenic differentiation of BMSCs	(94)
LINC00963	760	Inhibition of 760 expression	ETS1↑	downstream	Promoting osteogenic differentiation of BMSCs	(95)
XIST	19a-3p	Inhibition of 19a-3p expression	Hoxa5↑	downstream	Inhibiting osteogenic differentiation of BMSCs	(105)
XIST	29b-3p	Inhibition of 29b-3p expression	NNMT↑	downstream	Inhibiting osteogenic differentiation of BMSCs	(106)
HOTAIR	378g	Inhibition of 378g expression	NNMT↑	downstream	Inhibiting osteogenic differentiation of hBMSCs	(107)
DANCR	1301-3p	Inhibition of 1301-3p expression	PROX1↑	downstream	Inhibiting osteogenic differentiation of hBMSCs	(108)
SNHG1	101	Inhibition of 101 expression	DKK1↑	downstream	Inhibiting osteogenic differentiation of hBMSCs	(109)
HCG18	30a-5p	Inhibition of 30a-5p expression	Notch1↑	downstream	Inhibiting osteogenic differentiation of hBMSCs	(110)
MIAT	150-5p	Inhibition of 150-5p expression	MIAT↑	downstream	Inhibiting osteogenic differentiation of hBMSCs	(111)
BC083743	103-3p	Inhibition of 103-3p expression	SATB2↓	downstream	Inhibiting osteogenic differentiation of hBMSCs	(112)
MEG3	133a-3p	Promote the expression of 133a-3p	SLC39A1↓	downstream	Inhibiting osteogenic differentiation of hBMSCs	(113)
LNC-000052	96-5p	Inhibition of 96-5p expression	/	downstream	Inhibiting osteogenic differentiation of hBMSCs	(114)
H19	19b-3p	Inhibition of 19b-3p expression	/	downstream	Inhibiting osteogenic differentiation of hBMSCs	(115)

“/”: not have; “↑”: up-regulation; “↓”: down-regulation.

**Figure 7:**
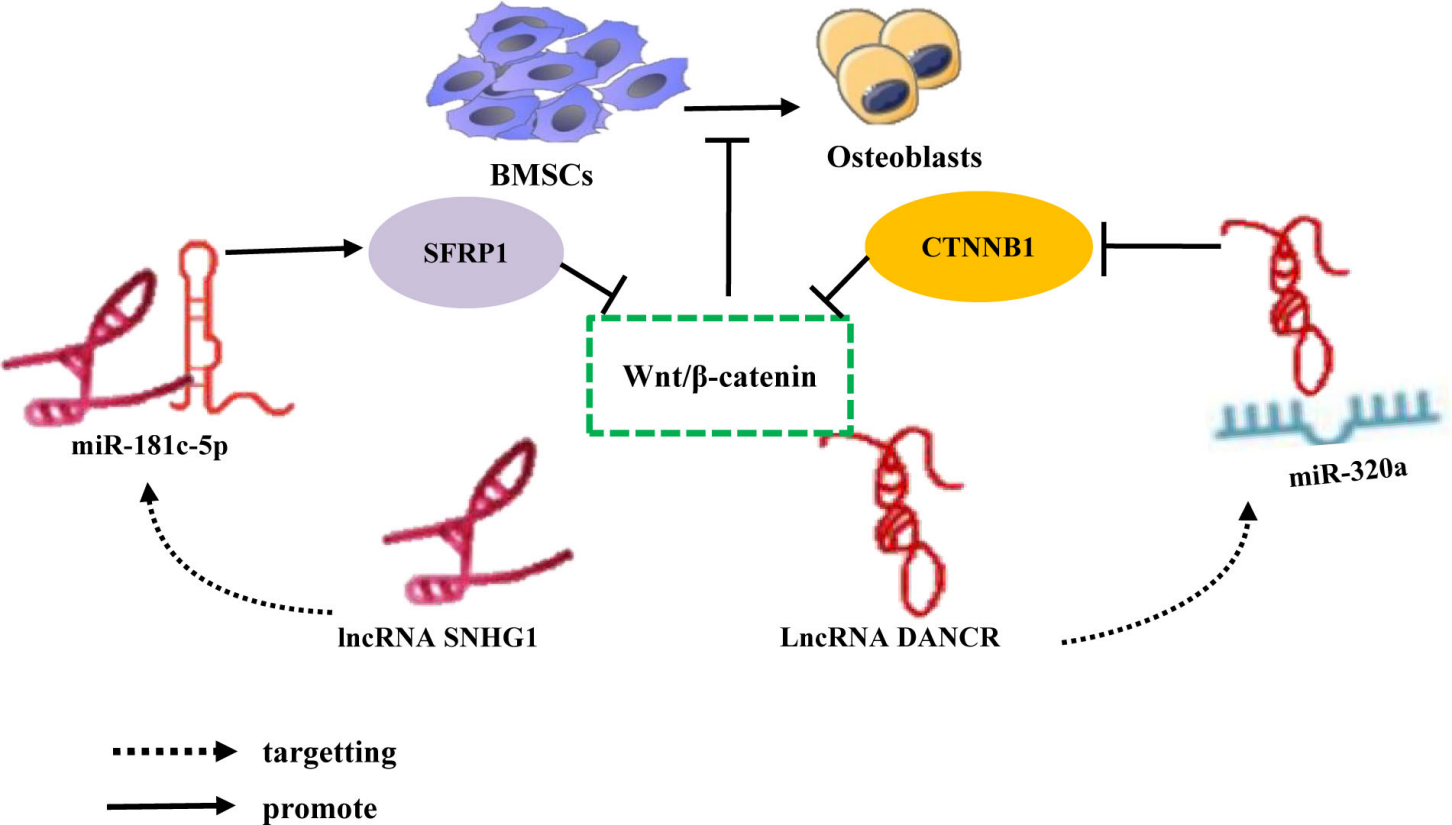
LncRNA-miRNA network regulates Wnt/β-Catenin signaling pathway inhibits osteogenic differentiation of BMSCs. Different lncRNAs bind to different miRNAs to inhibit miRNA expression, upregulate the downstream target gene SFRP1 expression of miRNA, inhibit the expression of CTNNB1, and inhibit Wnt/β-Catenin signaling pathway inhibits the osteogenic differentiation of BMSCs.

The Wnt/β-catenin signaling pathway is of significant importance in facilitating bone mineralization, governing the proliferation and differentiation of osteoblasts, promoting bone development, and maintaining bone homeostasis (116). Consequently, elucidating the precise regulatory mechanism of the Wnt/β-catenin signaling pathway in the differentiation of bone marrow-derived mesenchymal stem cells (BMSCs) is imperative for the maintenance of homeostasis. In recent years, studies have substantiated the substantial importance of the lncRNA-miRNA network loop in regulating the Wnt/β-catenin signaling pathway, particularly in relation to the differentiation of bone marrow-derived mesenchymal stem cells (BMSCs). For instance, Yu et al. conducted *in vivo* and *in vitro* experiments, which revealed an augmented expression of lncRNA SNHG1 in the serum and femoral tissue of OVX mice, accompanied by a reduction in miR-181c-5p. The elimination of SNHG1 was observed to enhance the osteogenic differentiation of BMSCs by upregulating miR-181c-5p. In contrast, the overexpression of SNHG1 was found to impede the osteogenic differentiation of BMSC. However, this inhibitory effect was counteracted when miR-181c-5p overexpressed. SNHG1 was observed to upregulate the expression of SFRP1 through its absorption of miR-181c-5p, which is known to act as a sponge for this microRNA. SFRP1 serves as an antagonist of the Wnt/β-catenin signaling pathway, which is responsible for inhibiting the downstream transduction of Wnt and its associated transduction proteins (117). Conversely, the overexpression of miR-181c-5p can activate the Wnt pathway by negatively regulating the expression of SFRP1 and promoting β-catenin signal transduction. It has been demonstrated that lncRNA SNHG1 acts as a sponge for miR-181c-5p, resulting in the upregulation of SFRP1 and the inhibition of Wnt/β-catenin signaling, thereby impeding the osteogenic differentiation of BMSCs (118). Furthermore, studies have revealed that lncRNA DANCR and miR-320a are upregulated, while CTNNB1 is downregulated in BMSCs of patients with osteoporosis. Notably, miR-320a and DANCR exert their functions independently, but the overexpression of either DANCR or miR-320a leads to a reduction in the expression levels of Wnt/β-catenin and CTNNB1, exhibiting additive effects. During the process of inducing osteogenic differentiation, the expression levels of DANCR and miR-320a exhibit a gradual decrease, whereas the expression level of CTNNB1 shows an increase. Nevertheless, it is noteworthy that miR-320a significantly inhibits the mRNA expression of CTNNB1, while miR-320a inhibitors promote its expression. This observation suggests a direct regulatory relationship between miR-320a and CTNNB1, which leads to a substantial downregulation of osteogenic differentiation and inhibition of the Wnt/β-catenin signaling pathway. Importantly, the inhibition of the catenin signaling pathway by miR-320a can be reversed, thereby counteracting one of its effects (**Table 2**; **Figure 7**) (119). The target, functioning as the recipient of information within the signal pathway, assumes a distinct role by interacting with specific molecules solely for the purpose of basic reception. To effectively transmit information, namely to propagate signals in both upstream and downstream directions, subsequently eliciting a sequence of responses and cascading reactions, the target necessitates the utilization of the signal pathway or signal axis. Previous studies have demonstrated that the Wnt/β-catenin signaling pathway, along with the PI3K/Akt/mTOR, OPG/RANK/RANKL, Notch, and Hedgehog signaling pathways, are intricately associated with the onset and progression of osteoporosis (120–125). However, studies regarding the impact of the lncRNA miRNA network loop on the regulation of these signaling pathways and its influence on the osteogenic differentiation of BMSCs remain unexplored in the existing literature. Based on the aforementioned study, the regulation of osteogenic differentiation in bone marrow-derived mesenchymal stem cells (BMSCs) is accomplished through the modulation of osteogenic-associated target genes, thereby establishing the lncRNA/miRNA/target gene axis or the lncRNA/miRNA/target gene/signaling pathway.

**Table 2:** LncRNA miRNA network regulates Wnt/β-Intervention of Catenin signaling pathway in osteogenic differentiation of bone marrow mesenchymal stem cells.

LncRNA	miRNA	Impact on miRNA	Impact on downstream target genes of miRNA	Signal access	Molecular function	Refs
H19	141	Inhibition of 141expression	β-catenin↑	Activate Wnt/ β-Catenin	Promoting osteogenic differentiation of hBMSCs	(99)
H19	22	Inhibition of 22expression	β-catenin↑	Activate Wnt/ β-Catenin	Promoting osteogenic differentiation of hBMSCs	(99)
ROR	138	Expression of antagonistic 138	ZEB2↑	Activate Wnt/ β-Catenin	Promoting osteogenic differentiation of MSCs	(100)
ROR	145	Expression of antagonistic 145	ZEB2↑	Activate Wnt/ β-Catenin	Promoting osteogenic differentiation of MSCs	(100)
C00707	145	Inhibition of 145 expression	LRP5↑	Activate Wnt/ β-Catenin	Promoting osteogenic differentiation of BMSCs	(101)
C00707	370-3p	Inhibition of 370-3p expression	Wnt2b↑	Activate Wnt/ β-Catenin	Promoting osteogenic differentiation of BMSCs	(102)
SNHG1	181c-5p	Inhibition of 181c-5p expression	SFRP1↑	Activate Wnt/ β-Catenin	Inhibiting osteogenic differentiation of BMSCs	(118)
DANCR	320a	Acting independently of each other	CTNNB1↓	Activate Wnt/ β-Catenin	Inhibiting osteogenic differentiation of hBMSCs	(119)

“↑”: up-regulation; “↓”: down-regulation.

## 4 The lncRNA–miRNA network regulates adipogenic differentiation of BMSCs

Various lncRNAs and miRNAs have been significantly correlated with adipogenic differentiation and play a crucial role in this process (126, 127). LncRNAs are important epigenetic regulatory factors that control gene expression and affect various biological processes, with potential regulatory effects on BMSC differentiation (111, 128). MiRNAs play a key role in the metabolic activity of bone cells; specifically, they can maintain bone metabolic homeostasis by regulating the phenotypic differentiation of BMSCs, which has potential therapeutic effects for metabolic bone diseases (129). BMSCs are important pluripotent stem cells and the main source of adipocytes throughout the body (13). Increasing evidence suggests that miRNAs are important regulatory targets of lncRNAs. LncRNAs play an important regulatory role in the adipogenic differentiation of BMSCs by acting as ceRNAs, which bind to miRNAs to inhibit their corresponding expression (130).

### 4.1 LncRNA–miRNA network regulation promotes adipogenic differentiation of BMSCs

**Figure 8:**
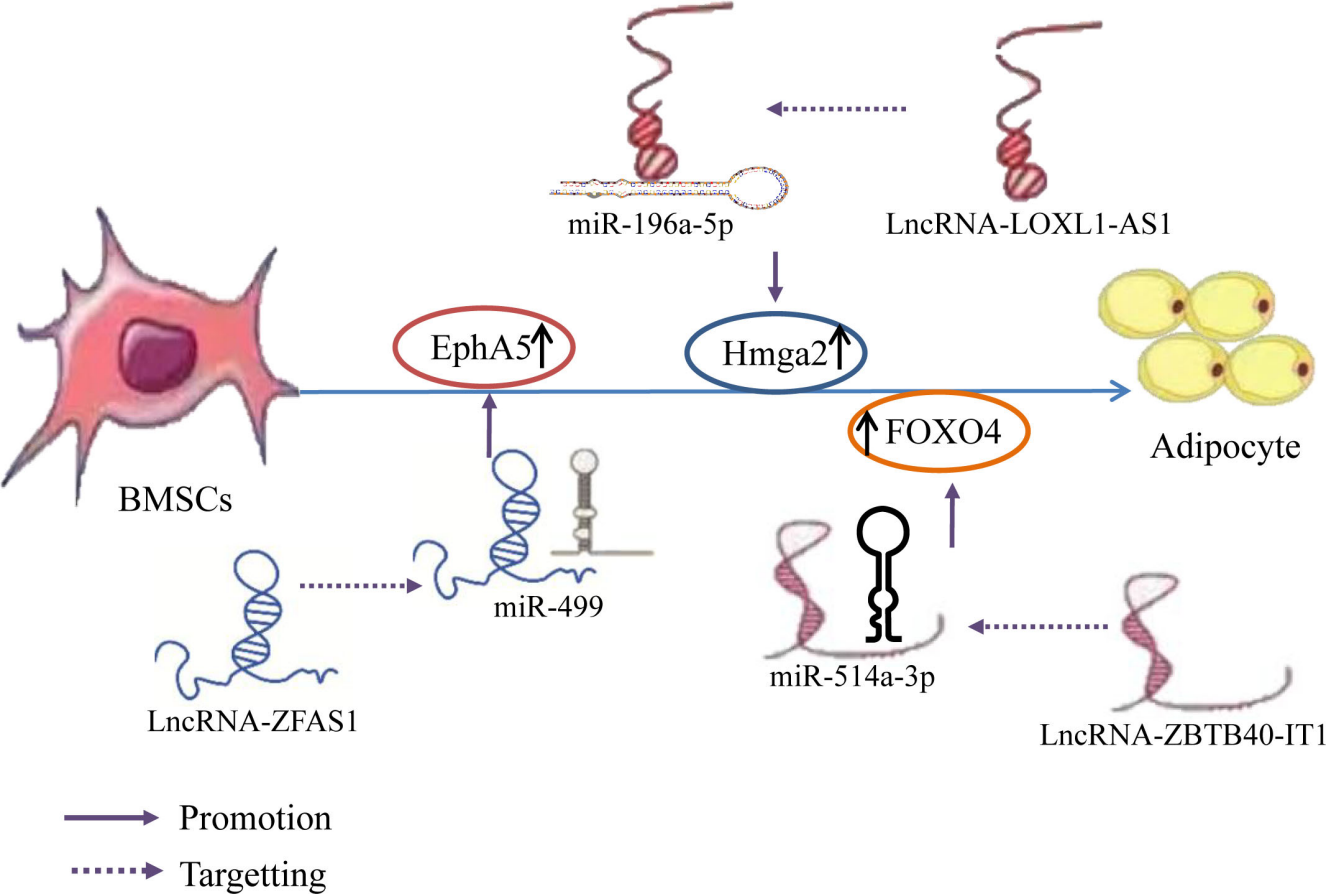
LncRNA–miRNA network regulation promotes adipogenic differentiation of BMSCs. Different lncRNAs bind to different miRNAs to inhibit miRNA expression and upregulate the expression of Hmga2, FOXO4, and EphA5, thereby promoting the adipogenic differentiation of hBMSCs.

It has been determined that during osteogenic differentiation of hBMSCs, the expression of lncRNAs LOXL1-AS1 and Hmga2 gradually decreases, while miR-196a-5p expression gradually increases. Overexpression of LOXL1-AS1 inhibits the osteogenic differentiation of hBMSCs, downregulates the expression level of miR-196a-5p, and promotes adipogenic differentiation. Alternatively, knocking down LOXL1-AS1 increases miR-196a-5p expression and inhibits adipogenic differentiation. This indicates that LOXL1-AS1 directly targets miR-196a-5p and negatively regulates its corresponding expression level. Hmga2 can activate C/EBPβ-mediated PPAR γ Expression to promote adipogenesis. Additionally, miR-196a-5p mimetics reduce Hmga2 expression, whereas miR-196a-5p inhibitors enhance Hmga2 expression. This indicates that Hmga2, as a target gene for miR-196a-5p, is positively correlated with LOXL1-AS1 and negatively correlated with miR-196a-5p. These results indicate that LOXL1-AS1 inhibits the miR-196a-5p expression and upregulates Hmga2 expression by binding to miR-196a-5p, thereby inhibiting the osteogenic differentiation of hBMSCs and promoting adipogenic differentiation (131). Some studies have also established that FOXO4 and the lncRNA ZBTB40-IT1 are downregulated during osteogenic differentiation of hBMSCs and upregulated during adipogenic differentiation. Knockdown, silencing, or overexpression of miR-514a-3p, which binds to ZBTB40-IT1 and FOXO4, promote osteogenic differentiation of hBMSCs but inhibit adipogenic differentiation; in contrast, miR-514a-3p inhibition reverses the effect of ZBTB40-IT1 knockdown on osteogenesis and adipogenesis of hBMSCs, indicating that the lncRNA ZBTB40-IT1 downregulates miR-514a-3p expression by directly binding to this miRNA. Additionally, overexpression of FOXO4 eliminates the effects of miR-514a-3p upregulation on hBMSC osteogenesis and adipogenesis. This indicates that lncRNA ZBTB40-IT1 inhibits the miR-514a-3p expression and positively regulates FOXO4 expression by binding to miR-514a-3p, thereby promotes adipogenic differentiation of hBMSCs (132). Further, Yan (133) et al. established that the lncRNA ZFAS1 was downregulated during BMSC osteogenic differentiation and upregulated during adipogenic differentiation, whereas miR-499 expression exhibited an opposite trend. A ZFAS1 knockout significantly promoted osteogenic differentiation and inhibited BMSC adipose differentiation, thereby enhancing miR-499 expression in BMSCs but reducing EphA5 expression. The knockout effect of ZFAS1 was weakened by co-transfection with miR-499 inhibitors. Overexpression of miR-499 significantly inhibited the mRNA and protein expression of EphA5 in BMSCs, indicating that lncRNA ZFAS1, as a ceRNA of miR-499, upregulated EphA5 expression by binding to miR-499, EphA5 is the downstream target gene of miR-49, thereby inhibiting the osteogenic differentiation of BMSCs and promoting their adipogenic differentiation. The results suggest that different lncRNAs bind to different miRNAs to inhibit miRNA expression and upregulate the expression of Hmga2, FOXO4, and EphA5, thereby promoting the adipogenic differentiation of hBMSCs (**Figure 8**).

### 4.2 LncRNA–miRNA network regulation inhibits adipogenic differentiation of BMSCs

**Figure 9:**
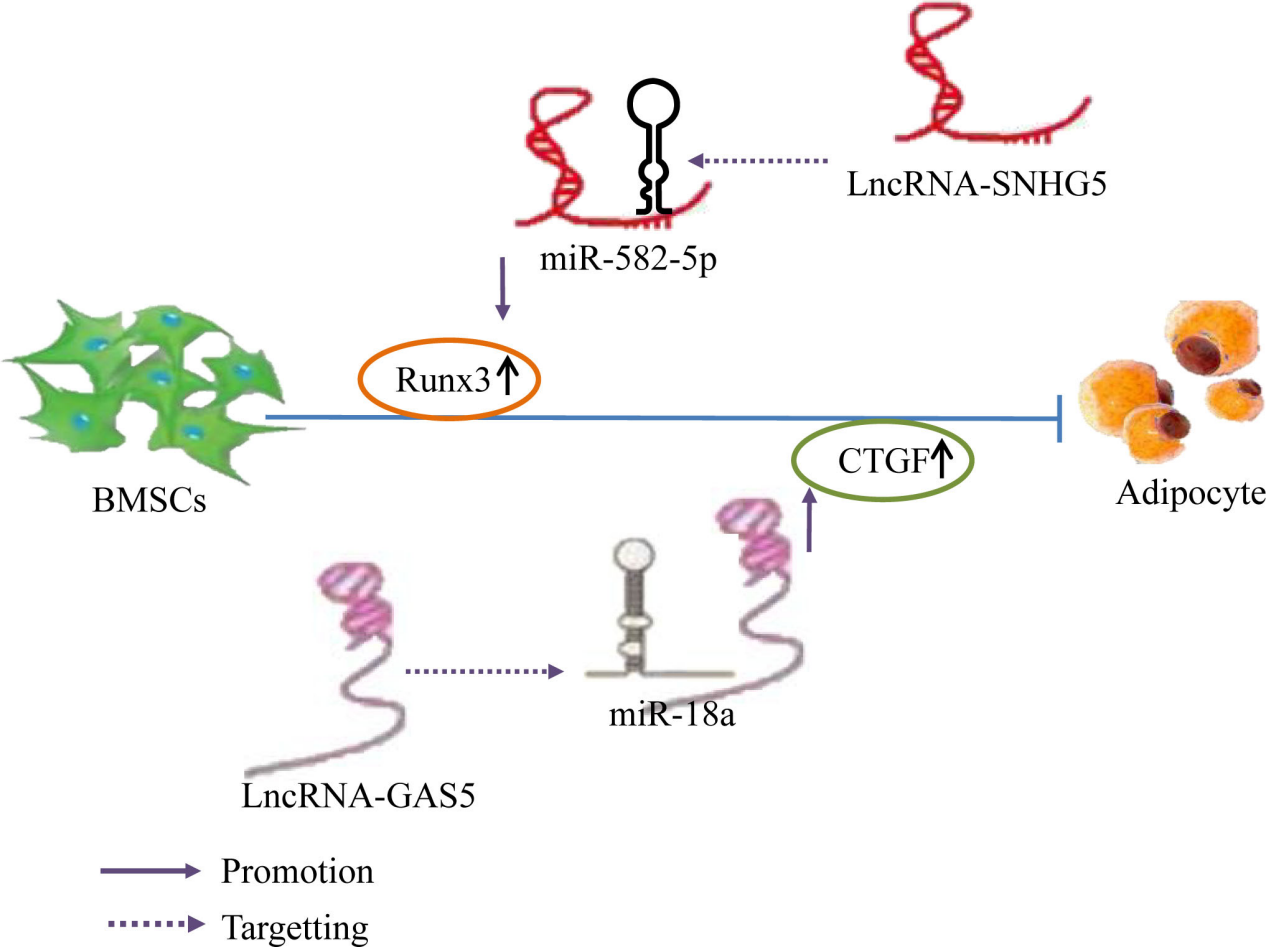
LncRNA–miRNA network regulation inhibits adipogenic differentiation of BMSCs. Different lncRNAs bind to different miRNAs to inhibit miRNA expression and upregulate the expression of Runx3 and CTGF, thereby inhibiting the adipogenic differentiation of hBMSCs.

Prior studies have found that the expression of Runx3 and the lncRNA SNHG5 gradually increase during the osteogenic differentiation and decrease during adipogenic differentiation of hBMSCs. Silencing of SNHG5 decreased Runx3 expression and significantly reduced alkaline phosphatase (ALP) activity during hBMSC osteogenic differentiation, inhibited osteogenic differentiation, and increased the expression levels of adipogenic markers. This effect could be restored by knocking down miR-582-5p or by upregulating Runx3. Silencing miRNA-582-5p expression in hBMSCs increased Runx3 expression; nonetheless, this effect was reversed by a SNHG5 knockout, indicating that lncRNA SNHG5 upregulates Runx3 expression by binding to miR-582-5p, Runx3 is the downstream target gene of miR-582-5p, promoting hBMSC osteogenic differentiation, and inhibiting adipogenic differentiation; this may be a novel mechanism for OP treatment (67). In addition, overexpression of the lncRNA GAS5 inhibits adipocyte formation in BMSCs and increases the CTGF protein expression, whereas knockdown of GAS5 has the opposite effect. In addition, miR-18a mimetics and inhibitors reversed the negative regulatory effect of GAS5 on the adipogenic differentiation of BMSCs. Further, miR-18a functions by binding to CTGF mRNA; consequently, CTGF protein expression decreases when BMSCs are treated with miR-18a mimetics and increases when BMSCs are treated with miR-18a inhibitors (134). This indicates that GAS5 inhibits the miR-18a expression and enhances CTGF protein translation by acting as an miR-18a sponge and ultimately inhibiting the adipogenic differentiation of BMSCs. Summary, Different lncRNAs bind to different miRNAs to inhibit miRNA expression and upregulate the expression of Runx3 and CTGF, thereby inhibiting the adipogenic differentiation of hBMSCs (**Figure 9**).

Downregulation of certain lncRNAs during the osteogenic differentiation of BMSCs negatively regulates the expression of miRNAs and their downstream molecules by targeting upregulated miRNAs; therefore, these lncRNAs play a role in inhibiting the osteogenic differentiation of BMSCs and promoting adipogenic differentiation. Additionally, the same lncRNA can exhibit different expression levels during BMSC osteogenic and adipogenic differentiation. By regulating the expression of miRNAs and their downstream molecules, osteogenic and adipogenic differentiation of BMSCs can be promoted or inhibited. The results are shown in **Table 3**. CircRNA, lncRNA and miRNA belong to the same family: non-coding RNAs. However, there has been limited research on the circRNA–miRNA network in BMSC osteogenesis and differentiation; additionally, there is no prior report discussing the current research on the effects of circRNA–miRNA mechanisms on BMSC adipogenesis and differentiation. Therefore, the circRNA–miRNA-mediated regulation of BMSC adipogenesis and differentiation may be a future field of interest.

**Table 3:** LncRNA miRNA network regulates adipogenic differentiation of bone marrow mesenchymal stem cells.

LncRNA	miRNA	Impact on miRNA	Impact on downstream target genes of miRNA	The relationship between target genes and networks	Molecular function	Refs
LOXL1-AS1	196a-5p	Inhibition of 196a-5p expression	Hmga2↑	downstream	Promoting adipogenic differentiation of hBMSCs	(131)
ZBTB40-IT1	514a-3p	Inhibition of 217 expression	FOXO4↑	downstream	Promoting adipogenic differentiation of hBMSCs	(132)
ZFAS1	499	Inhibition of 498 expression	EphA5↑	downstream	Promoting adipogenic differentiation of hBMSCs	(133)
SNHG5	582-5p	Inhibition of 582-5p expression	Runx3↑	downstream	Inhibition of adipogenic differentiation of hBMSCs	(67)
GAS5	18a	Inhibition of 18a expression	CTGF↑	downstream	Inhibition of adipogenic differentiation of hBMSCs	(134)

“↑”: up-regulation; “↓”: down-regulation.

## 5 The potential therapeutic effect of drugs targeting the lncRNA–miRNA network to regulate osteogenic/adipogenic differentiation of BMSCs

BMSCs can self-renew and differentiate into bone cells; therefore, BMSCs play an important role in the regulation of bone homeostasis (6). However, under pathological and physiological conditions, such as aging, OP, and bone defects, the differentiation ability of BMSCs can be disrupted (135). Therefore, it is necessary to develop drugs that can regulate the osteogenic/adipogenic differentiation of BMSCs to treat these orthopaedic diseases.

### 5.1 Hormones

**Figure 10:**
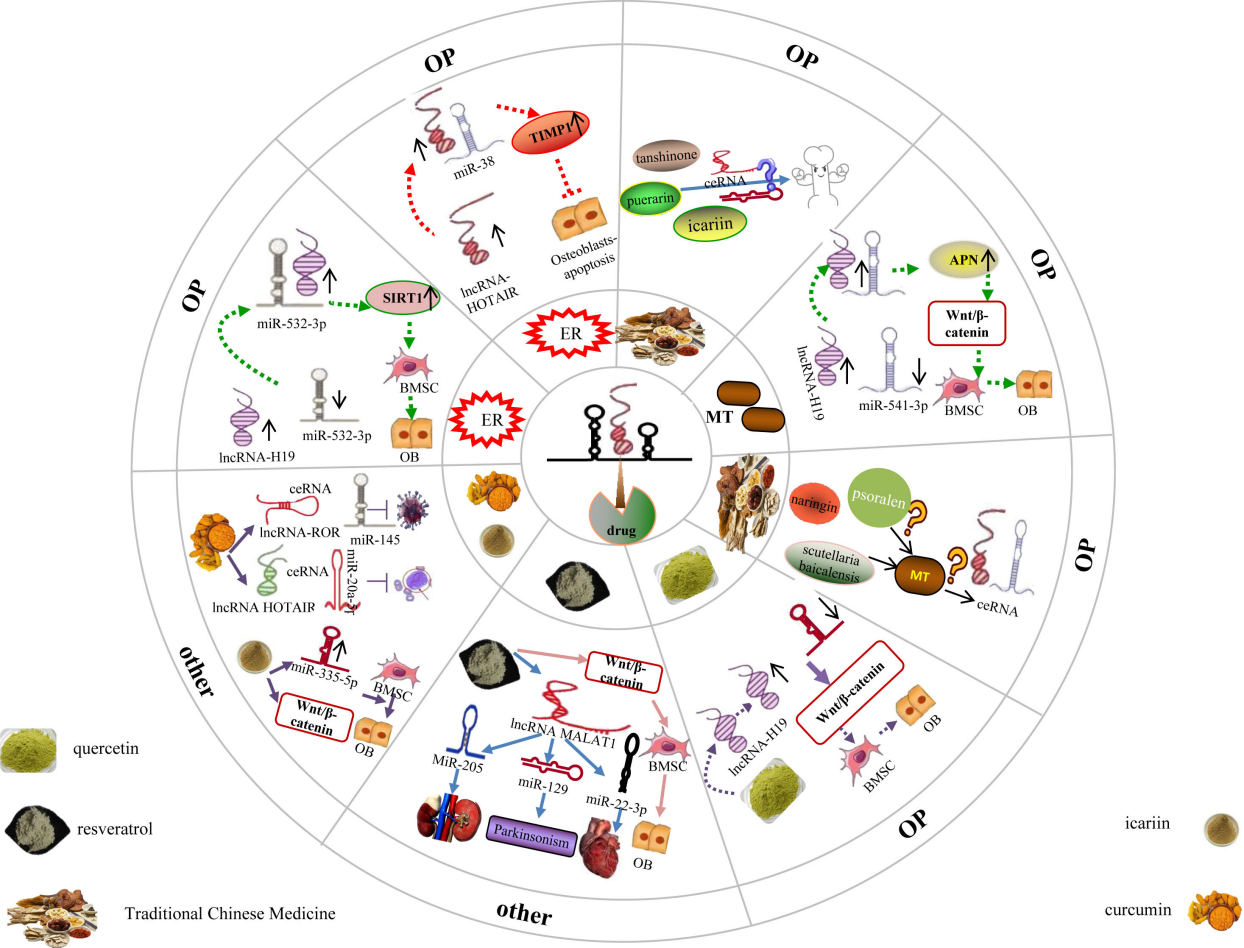
Drug targeted lncRNA-miRNA network regulates BMSC to promote bone lipid differentiation in BMSC. Different hormones and traditional Chinese medicine have played a therapeutic role in treating OP by targeting and regulating the lncRNA miRNA network loop, while it is still unclear whether some traditional Chinese medicine that can improve these hormones can also exert the same effect through the lncRNA miRNA network loop. In addition, studies have confirmed that some traditional Chinese medicine with therapeutic effects on OP can exert therapeutic effects in other diseases through the lncRNA miRNA network ring, but it is still unclear whether it exerts therapeutic effects on OP through this network ring.

The hormone status significantly governs and exerts a substantial impact on the health and overall well-being of women. The absence of estrogen and the disruption in age-related hormone levels contribute to the development of diverse diseases, thereby influencing the advancement of age-related ailments in women, including osteoporosis (136). Nevertheless, the precise mechanism underlying hormone therapy for osteoporosis remains elusive. Recent studies have revealed that estrogen modulates the lncRNA miRNA network loop, thereby potentially playing a role in the treatment of osteoporosis. Li et al. (27) conducted discovered that the expression of lncRNA H19 and SIRT1 was downregulated, while miR-532-3p expression was upregulated in both postmenopausal osteoporosis (PMOP) patients and ovariectomized (OVX) rats *in vivo*. However, after estrogen intervention, this outcome was reversed. Additionally, the study revealed that the upregulated lncRNA H19 directly binds to miR-532-3p, inhibiting its expression, while simultaneously upregulating the downstream target gene SIRT1 expression of miR-532-3p. Consequently, this process promotes osteogenic differentiation of bone marrow mesenchymal stem cells (BMSCs). The study by Xu et al. (137) demonstrates that estrogen is a key factor in the osteogenic differentiation of bone marrow-derived mesenchymal stem cells (BMSCs) through the involvement of the long non-coding RNA (lncRNA) H19/miR-532-3p/SIRT1 axis. This finding provides valuable insights into the potential treatment of postmenopausal osteoporosis (PMOP). Additionally, the researchers observed a downregulation of the lncRNA HOTAIR in postmenopausal women, which can bind to miR-138 and negatively regulate its expression. Furthermore, miR-138 can bind to its target gene TIMP1 and reduce its expression. Following estrogen intervention, a dose-dependent increase in the expression of the long non-coding RNA (lncRNA) HOTAIR was observed. This upregulation of HOTAIR subsequently led to elevated levels of TIMP1 and enhanced viability of osteoblasts by targeting miR-138 and inhibiting osteoblast apoptosis. The inhibition of HOTAIR expression effectively blocked this estrogen-induced effect. These findings suggest that estrogen induces the upregulation of lncRNA HOTAIR, which in turn targets the binding of miR-138 and upregulates the expression of TIMP1. Ultimately, this signaling axis involving lncRNA HOTAIR/miR-138/TIMP1 plays a therapeutic role in osteoporosis by inhibiting osteoblast apoptosis in postmenopausal women.The aforementioned findings provide evidence that the modulation of bone marrow-derived mesenchymal stem cell (BMSC) lipid differentiation via ER targeting of the lncRNA-miRNA network loop can elicit an anti-OP effect (**Figure 10**). It has been established through previous research (138–140) that Puerarin, icariin, tanshinone, and other active ingredients found in traditional Chinese medicine possess the ability to enhance estrogen levels. However, the potential impact of these substances on the regulation of the lncRNA miRNA network loop, their interference with bone marrow mesenchymal stem cell (BMSC) differentiation into adipocytes, and their therapeutic role in the treatment of osteoporosis (OP) has not been reported (**Figure 10**).

Furthermore, several studies have indicated a significant correlation between the alteration of Melatonin (MT) and the onset and progression of osteoporosis (OP) (141). Melatonin (MT), a methoxyindole, is primarily synthesised and secreted by the pineal gland at night under normal light and dark conditions; additionally, MT can be synthesised in the mitochondria (142), which indicates that every cell, including BMSCs and osteoblasts, can synthesise MT. MT not only regulates the circadian rhythm (143), but also has antioxidant (144), anti-aging (145), and immune-regulatory properties (146). Furthermore, it is worth noting that MT is closely associated with bone metabolism homeostasis, and its reduced expression is a key factor in bone loss and OP (147, 148). The vivo research finds that the MT, lncRNA H19, and aminopeptidase N (APN) expression were downregulated, whereas miR-541-3p expression was upregulated in an ovariectomized (OVX) rat model. At the cellular level, MT inhibits adipogenic differentiation of BMSCs, promotes osteogenic differentiation of BMSCs, and activates the Wnt/β-catenin pathway, which is reversed by MT inhibitors. Additionally, H19 overexpression promotes MT-mediated osteogenic differentiation of BMSCs, inhibits adipogenic differentiation, reduces miR-541-3p levels, and increases APN expression. Contrastingly, H19 knockdown or miR-541-3p overexpression has the opposite effect. In addition, H19 promotes the osteogenic differentiation of BMSCs by binding to miR-541-3p, an miRNA that targets APN, an adipocyte-specific factor (149). This indicates that MT upregulates lncRNA H19 and inhibits miR-541-3p expression, upregulates the APN expression in turn, APN is the downstream target gene of miR-541-3p, thereby inhibiting BMSC adipogenic differentiation and enhancing their osteogenic differentiation by activating the Wnt/β-catenin pathway. In summary, MT provides a new reference for the targeted treatment of OP (**Figure 10**).

With the development of network pharmacology and molecular docking technology, These studies have also demonstrated the efficacy of Naringin, Psoralen, and other active ingredients derived from traditional Chinese medicine in promoting bone preservation and treating OP (150, 151). Additionally, Melatonin has been identified in numerous medicinal plants, including scutellaria baicalensis (152), most of which possess cooling properties, a bitter taste, and are associated with the liver meridian. Currently, there is a lack of research reports investigating the potential impact of a specific Chinese medicine with osteoprotective properties and Fahui, the active ingredient of Chinese medicine, on osteoporosis (OP). Specifically, it remains unclear whether this effect is achieved through the enhancement of Melatonin levels, subsequently intervening in the lncRNA miRNA network loop to facilitate osteogenic differentiation and impede adipogenic differentiation of bone marrow mesenchymal stem cells (BMSCs). The aforementioned research demonstrates that hormones have the capacity to enhance the osteogenic differentiation of bone marrow-derived mesenchymal stem cells (BMSC) through the modulation of the lncRNA miRNA network circuitry. In addition, traditional Chinese medicine has been found to augment the expression of estrogen, melatonin, and other hormones. Is it plausible to hypothesize that traditional Chinese medicine (naringin, psoralen, scutellaria baicalensis) can indirectly modulate the lncRNA miRNA network loop through the regulation of hormone expression, subsequently influencing the downstream target gene expression of miRNA, thereby modulating the differentiation of bone marrow mesenchymal stem cells (BMSC) into adipocytes and preventing osteoporosis (OP) by intervening in pertinent signaling pathways? This warrants further research (**Figure 10**).

### 5.2 Effective ingredients of traditional Chinese medicine

Previous studies have found that quercetin, a natural flavonoid, has antioxidant and anti-inflammatory effects; additionally, this well-known phytoestrogen plays an important role in the osteogenic differentiation of BMSCs (153, 154). Quercetin can enhance ALP activity and upregulate

the expression of BMP2, osteocalcin, and Runx2, thus promoting the osteogenic differentiation of BMSCs. Additionally, lncRNA H19 promotes the osteogenic differentiation of BMSCs, miR-625-5p inhibits the osteogenic differentiation of BMSCs, and quercetin increases lncRNA H19 expression; nonetheless, the quercetin-mediated effects on BMSCs could be reversed by silencing H19 expression. Additionally, silencing miR-625-5p promotes the proliferation and osteogenic differentiation of BMSCs, whereas overexpression of miR-625-5p reverses the quercetin-mediated osteogenic differentiation of BMSCs. Further, quercetin treatment or miR-625-5p downregulation increases β-Catenin protein levels (155), indicating that quercetin activates Wnt via the lncRNA H19-miR-625-5p network/β-catenin pathway, ultimately promoting BMSC proliferation and osteogenic differentiation, delaying occurrence of bone loss, and presenting novel targets and ideas for the therapeutic management of osteoporosis (**Figure 10**).

Resveratrol, a natural polyphenol extracted from plants, not only has no toxic effects but also possesses various beneficial pharmacological effects. Specifically, resveratrol can alter the expression of intracellular mediators, regulate the cell cycle, metabolism, post-translational modifications, and inflammatory responses (156), and plays an important role in antitumour, anti-inflammatory, and oxygen free radical scavenging mechanisms (157). For example, resveratrol targets different miRNAs such as miR-205, miR-129/SNCA, and miR-22-3p via lncRNA MALAT1, thereby alleviating acute kidney injury caused by sepsis (158), improving the Parkinson’s disease-like phenotype (159), and alleviating heart damage associated with pulmonary embolism (160), respectively; therefore, resveratrol may be a strong candidate for the treatment of several diseases. Recent studies have shown that resveratrol can activate the Wnt/β-catenin signalling pathway to promote osteogenic differentiation and prevent bone loss in mouse and hBMSCs (161). However, it is yet to be determined whether resveratrol exerts this effect by mediating the lncRNA**–**miRNA network. In addition, icariin is the main active flavonoid glycoside of Epimedium; this flavonoid has been demonstrated to possess therapeutic effects in OVX rat models and in postmenopausal women with OP. Additionally, Teng (162) et al. determined that icariin mediates osteogenic differentiation of BMSCs via miR-335-5p upregulation. Other studies established that icariin activates the Wnt/β-catenin signalling pathway and promotes the osteogenic differentiation of BMSCs (163). However, a comprehensive study regarding the potential of icariin to enhance osteogenic differentiation, modulate bone metabolism, and serve as a therapeutic intervention for osteoporosis in bone marrow mesenchymal stem cells (BMSCs) through the regulation of the lncRNA miRNA network loop and activation of the Wnt/catenin signaling pathway is currently lacking. Further, as a yellow natural polyphenol compound extracted from the curcumin rhizome, icariin can mediate the lncRNA**–**miRNA network, which could be used for the treatment of various diseases. For example, curcumin inhibits the proliferation and invasion of human prostate cancer stem cells *in vitro* via the corresponding ceRNA effects of miR-145 and lncRNA-ROR (164); additionally, curcumin reduces the Adriamycin resistance of acute myeloid leukaemia by inhibiting the lncRNA HOTAIR/miR-20a-5p/WT1 axis (165). Overall, the therapeutic potential of curcumin for the treatment age-related diseases (including OP) has been fully demonstrated. For example, Kim (166, 167) et al. confirmed the protective effects of curcumin on OVX-induced bone loss; specifically, this may be attributed to an increase in the expression of osteoblast differentiation-related genes, leading to an increase in ALP activity and mineralisation. Furthermore, the regulation of the lncRNA-miRNA network ring by Resveratrol, icariin, Curcumin, and other monomers found in traditional Chinese medicine is shown to support their therapeutic effects on various diseases.

The above results indicate that the lncRNA miRNA network loop is an important target for disease treatment, However, the potential involvement of Resveratrol, icariin, and Curcumin, which are frequently utilized monomers in bone, in the signaling pathway associated with bone adipogenesis, their ability to impede the differentiation of bone marrow-derived mesenchymal stem cells (BMSC) into adipocytes, their capacity to regulate bone homeostasis, prevent bone loss, and potentially treat osteoporosis (OP) by modulating the lncRNA miRNA network, remains unexplored in the existing literature. Consequently, further comprehensive studies are warranted by scholars to generate novel insights for future OP therapeutics (**Figure 10**).

## 6 Summary and outlook

OP, a systemic metabolic disease, is distinguished by disrupted bone homeostasis. The presence of bone marrow-derived mesenchymal stem cells (BMSC) with the capacity for multi-directional differentiation and self-renewal plays a vital role in maintaining bone homeostasis. BMSC have the ability to differentiate into osteoblasts and adipocytes. In normal physiological conditions, the differentiation of BMSC into osteoblasts and adipocytes is mutually regulated, maintaining a dynamic equilibrium. However, when exposed to pathological factors, the differentiation of BMSC into adipocytes surpasses that into osteoblasts, leading to abnormal bone fat differentiation and an imbalance in bone homeostasis. An increasing body of evidence indicates that OP arises from a decline in osteogenesis and an increase in adipogenesis. Consequently, the promotion of osteoblast differentiation and the inhibition of adipocyte differentiation hold significant importance in restoring proper bone homeostasis, thereby offering potential therapeutic avenues for OP treatment. In recent years, the “LncRNA miRNA” network loop has gained significant attention in current research due to advancements in network pharmacology and Macromolecular docking technology. LncRNA, functioning as a ceRNA, exerts a substantial influence on disease prevention and treatment by inhibiting miRNA expression through sponge adsorption and subsequently regulating downstream target genes. Furthermore, the comprehensive demonstration of the involvement of the “LncRNA miRNA” network loop in the treatment of OP has been established through its regulation of bone marrow mesenchymal stem cell (BMSC) differentiation into adipocytes and the maintenance of bone homeostasis (168–170). Moreover, it has been observed that certain traditional Chinese medicines, known for their therapeutic effects on OP, can also modulate the “LncRNA miRNA” network loop and influence BMSC’s differentiation into adipocytes, suggesting that the therapeutic efficacy of traditional Chinese medicine in OP may be attributed to its impact on the “LncRNA miRNA” network loop. However, there is a dearth of scholarly research pertaining to the utilization of traditional Chinese medicine as an intervention in the treatment of osteoporosis through the regulation of the “LncRNA miRNA” network loop in the differentiation of bone marrow mesenchymal stem cells (BMSCs) into adipocytes. Consequently, there exists substantial scope for further advancements in this particular field of study. Furthermore, it is widely acknowledged that the onset and progression of osteoporosis are not solely contingent upon the equilibrium of bone marrow mesenchymal stem cell-mediated bone fat regulation, but also intricately linked to the uncoupling of osteoblasts and osteoclasts. Bone remodeling is a complex process that relies on the coordinated actions of osteoblasts for bone formation and osteoclasts for bone resorption. Osteoclasts, which originate from mononuclear macrophages and are characterized by their multinucleated nature, play a crucial role in the regulation of bone remodeling. Any abnormalities in their differentiation or dysfunction can contribute to the development of various bone diseases, such as osteoporosis (OP). Consequently, it is of utmost importance to investigate the means and mechanisms by which osteoclasts can be regulated, as this knowledge may lead to the identification of potential therapeutic targets for the treatment of OP. The occurrence and progression of osteoporosis (OP) are widely acknowledged to be influenced by the regulation of bone fat balance by bone marrow stromal cells (BMSC), as well as the decoupling of osteoblasts and osteoclasts. Given that bone remodeling is a dynamic process, it necessitates the coordinated actions of osteoblasts for bone formation and osteoclasts for bone resorption in order to maintain its integrity. Osteoclasts, which originate from mononuclear macrophages and are characterized by their multinucleated structure specific to bone tissue, play a crucial role in the intricate process of bone remodeling. The differentiation and malfunction of these cells can give rise to a range of bone disorders, such as OP. Consequently, it is imperative to investigate the means and mechanisms by which osteoclasts can be regulated, in order to identify potential therapeutic targets for the treatment of OP. In recent years, researches have revealed that the network loop involving long non-coding RNA (lncRNA) and microRNA (miRNA) not only governs the differentiation of bone lipids in bone marrow-derived mesenchymal stem cells (BMSC), thereby sustaining bone homeostasis and addressing osteoporosis (OP), but also assumes a significant function in the prevention and treatment of OP through the regulation of osteoclast differentiation in monocytes and macrophages. For instance, during the process of osteoclast differentiation of monocyte macrophages, lncRNA Xist and lncRNA Neat1 function as competing endogenous RNAs (ceRNAs) for miR-590-3p and miR-7, respectively. This interaction leads to the upregulation of Tgif2 and PTK2, downstream target genes of miR-590-3p and miR-7, thereby promoting the differentiation and maturation of osteoclasts (171, 172). Additionally, lncRNA MIRG acts as a ceRNA for miR-1897, sequestering it and resulting in the upregulation of its co-expression gene NFATc1. This process induces the formation of osteoclasts and bone resorption in patients with osteoporosis (173). Furthermore, several studies (174) have demonstrated that lncRNA-MALAT1 exerts a negative control on miR-124 activity through direct targeting, while also promoting osteoclast differentiation. These findings highlight the significance of the lncRNA miRNA network loop in not only regulating bone lipid differentiation and bone homeostasis, but also in playing a crucial role in the regulation of the osteogenic osteoclast coupling balance mechanism. However, the majority of research studies have primarily concentrated on investigating the facilitation of osteoclast differentiation through the lncRNA miRNA network loop in monocytes and macrophages, while limited attention has been given to exploring the suppression of osteoclast differentiation. Furthermore, non coding RNA encompasses lncRNA, miRNA, and circRNA. Currently, researches are being conducted on the circRNA-miRNA network loop, which has the potential to enhance the osteogenic differentiation of BMSC by modulating the osteogenic differentiation of BMSC (175), as well as the maturation and differentiation of osteoclasts in the osteogenic osteoclast coupling mechanism (176, 177). Additionally, drug intervention within this network loop is being explored as a means to prevent and treat OP (175). However, there is a lack of pertinent literature regarding the study of BMSC adipogenic differentiation and drug intervention in the management of mononuclear macrophage osteoclast differentiation through this network loop. Hence, the network loop involving lncRNA-miRNA-circRNA plays a regulatory role in the differentiation of bone marrow-derived mesenchymal stem cells (BMSCs) into bone fat cells and monocyte-derived macrophages into osteoclasts. Moreover, traditional Chinese medicine interventions in this network loop have the potential to modulate BMSC bone fat differentiation and suppress monocyte macrophage osteoclast differentiation, thereby preserving bone homeostasis. This phenomenon presents a promising avenue for future osteoporosis treatment and merits further research and scholarly inquiry.

## Author contributions

FA and XW wrote the article. CW, YL, BS, JZ, and PG drew the figures of the manuscript. CY proposed the conception or design of the work All. authors edited and check the final manuscript. All authors contributed to the article and approved the submitted version.

## References

[1] Gibon E Lu LY Nathan K Goodman SB. Inflammation, ageing, and bone regeneration. J Orthop Translat (2017) 10:28–35. doi: 10.1016/j.jot.2017.04.002 29094003 PMC5662134

[2] Gu H Shi S Xiao F Huang Z Xu J Chen G. MiR-1-3p regulates the differentiation of mesenchymal stem cells to prevent osteoporosis by targeting secreted frizzled-related protein 1. Bone (2020) 137:115444. doi: 10.1016/j.bone.2020.115444 32447074

[3] Dall TM Gallo PD Chakrabarti R West T Semilla AP Storm MV. An aging population and growing disease burden will require a large and specialized health care workforce by 2025. Health Aff (Millwood) (2013) 32(11):2013–20. doi: 10.1377/hlthaff.2013.0714 24191094

[4] Li X Wu J Liu S Zhang K Miao X Li J. miR-384-5p targets Gli2 and negatively regulates age-related osteogenic differentiation of rat bone mar-row mesenchymal stem cells. Stem Cells Dev (2019) 28(12):791–8. doi: 10.1089/scd.2019.0044 30950325

[5] Li M Yang N Hao L Zhou W Li L Liu L. Melatonin inhibits the ferroptosis pathway in rat bone marrow mesenchymal stem cells by activating the PI3K/AKT/mTOR signaling axis to attenuate steroid-induced osteopo-rosis. Oxid Med Cell Longev (2022) 2022:8223737. doi: 10.1155/2022/8223737 36035224 PMC9410838

[6] Luby AO Ranganathan K Lynn JV Nelson NS Donneys A Buchman SR. Stem cells for bone regeneration: current state and future directions. J Craniofac Surg (2019) 30(3):730–5. doi: 10.1097/SCS.0000000000005250 30817525

[7] Li N Liu L Liu Y Luo S Song Y Fang B. miR-144-3p suppresses osteogenic differentiation of BMSCs from patients with aplastic anemia through repression of TET2. Mol Ther Nucleic Acids (2020) 19:619–26. doi: 10.1016/j.omtn.2019.12.017 PMC6965517 31945725

[8] Ning K Liu S Yang B Wang R Man G Wang DE. Update on the effects of energy metabolism in bone marr-ow mesenchymal stem cells differentiation. Mol Metab (2022) 58:101450. doi: 10.1016/j.molmet.2022.101450 35121170 PMC8888956

[9] Zhi F Ding Y Wang R Yang Y Luo K Hua F. Exosomal hsa_circ_0006859 is a potential biomarker for postmenopausal osteoporosis and enhances adipogenic versus osteogenic differentiation in human bone marrow mesenchymal stem cells by sponging miR-431-5p. Stem Cell Res Ther (2021) 12(1):157. doi: 10.1186/s13287-021-02214-y 33648601 PMC7923524

[10] Zhang L Li S Li J Li Y. LncRNA ORLNC1 promotes bone marrow mesenchyml stem cell pyroptosis induced by advanced glycation end production by targeting miR-200b-3p/foxo3 pathway. Stem Cell Rev Rep (2021) 17(6):2262–75. doi: 10.1007/s12015-021-10247-2 34482528

[11] He M Lei H He X Liu Y Wang A Ren Z. METTL14 Regulates Osteogenesis of Bone Marrow Mesenchymal Stem Cells via Inducing Autophagy Through m6A/ IGF2BPs/Beclin-1 Signal Axis. Stem Cells Transl Med (2022) 11(9):987–1001. doi: 10.1093/stcltm/szac049 35980318 PMC9492283

[12] Lin Z He H Wang M Liang J. MicroRNA-130a controls bone marrow mesenchymal stem cell differentiation towards the osteoblastic and adipogenic fate. Cell Prolif (2019) 52(6):e12688. doi: 10.1111/cpr.12688 31557368 PMC6869834

[13] Qi M Zhang L Ma Y Shuai Y Li L Luo K. Autophagy maintains the function of bone marrow mesenchymal stem cells to prevent estrogen deficiency-induced osteoporosis. Theranostics (2017) 7(18):4498–516. doi: 10.7150/thno.17949 PMC5695145 29158841

[14] Hu L Yin C Zhao F Ali A Ma J Qian A. Mesenchymal stem cells: cell fate decision to osteoblast or adipocyte and application in osteoporosis treatment. Int J Mol Sci (2018) 19(2):360. doi: 10.3390/ijms19020360 29370110 PMC5855582

[15] Li H Liu P Xu S Li Y Dekker JD Li B. FOXP1 controls mesenchymal stem cell commitment and senescence during skeletal aging. J Clin Invest (2017) 127(4):1241–53. doi: 10.1172/JCI89511 PMC5373872 28240601

[16] Qadir A Liang S Wu Z Chen Z Hu L Qian A. Senile osteoporosis: the involvement of differentiation and senescence of bone marrow stromal cells. Int J Mol Sci (2020) 21(1):349. doi: 10.3390/ijms21010349 31948061 PMC6981793

[17] Pokrovskaya LA Nadezhdin SV Zubareva EV Burda YE Gnezdyukova ES. Expression of RUNX2 and Osterix in Rat Mesenchymal Stem Cells during Culturing in Osteogenic-Conditio- ned Medium. Bull Exp Biol Med (2020) 169(4):571–5. doi: 10.1007/s10517-020-04931-5 32910396

[18] Hong S Hu S Kang Z Liu Z Yang W Zhang Y. Identification of functional lncRNAs based on competing endogenous RNA network in osteoblast differentiation. J Cell Physiol (2020) 235(3):2232–44. doi: 10.1002/jcp.29132 31486078

[19] Huang W Wu Y Qiao M Xie Z Cen X Huang X. CircRNA-miRNA networks in regulating bone disease. J Cell Physiol (2022) 237(2):1225–44. doi: 10.1002/jcp.30625 34796958

[20] Mandourah AY Ranganath L Barraclough R Vinjamuri S Hof RV Hamill S. Circulating microRNAs as potential diagnostic biomarkers for osteoporosis. Sci Rep (2018) 8(1):8421. doi: 10.1038/s41598-018-26525-y 29849050 PMC5976644

[21] Zhang J Hao X Yin M Xu T Guo F. Long non-coding RNA in osteogenesis: A new world to be explored. Bone Joint Res (2019) 8(2):73–80. doi: 10.1302/2046-3758.82.BJR-2018-0074.R1 30915213 PMC6397330

[22] Hodges WM O'Brien F Fulzele S Hamrick MW. Function of microRNAs in the osteogenic diffe-rentiation and therapeutic application of adipose-derived stem cells (ASCs). Int J Mol Sci (2017) 18(12):2597. doi: 10.3390/ijms18122597 29207475 PMC5751200

[23] Wang J Liu S Li J Zhao S Yi Z. Roles for miRNAs in osteogenic differentiation of bone marrow mesenchymal stem cells. Stem Cell Res Ther (2019) 10(1):197. doi: 10.1186/s13287-019-1309-7 31253175 PMC6599379

[24] Wei C Sun Y Wang J Lin D Cui V Shi H. NONMMUT055714 acts as the sponge of micro-RNA-7684-5p to protect against postoperative cognitive dysfunction. Aging (Albany NY) (2021) 13(9):12552–64. doi: 10.18632/aging.202932 PMC8148455 33902009

[25] Li K Wu Y Yang H Hong P Fang X Hu Y. H19/miR-30a/C8orf4 axis modulates the adipogenic differentiation process in human adipose tissue-derived mesenchymal stem cells. J Cell Physiol (2019) 234(11):20925–34. doi: 10.1002/jcp.28697 31026067

[26] Bian M Yu Y Li Y Zhou Z Wu X Ye X. Upregulating the Expression of LncRNA ANRIL Promotes Osteogenesis via the miR-7-5p/IGF-1R Axis in the Inflamed Periodontal Ligament Stem Cells. Front Cell Dev Biol (2021) 9:604400. doi: 10.3389/fcell.2021.604400 33692995 PMC7937634

[27] Li T Jiang H Li Y Zhao X Ding H. Estrogen promotes lncRNA H19 expression to regulate osteogenic differentiation of BMSCs and reduce osteoporosis via miR-532-3p/SIRT1 axis. Mol Cell Endocrinol (2021) 527:111171. doi: 10.1016/j.mce.2021.111171 33577975

[28] Bhan A Soleimani M Mandal SS. Long noncoding RNA and cancer: A new paradigm. Cancer Res (2017) 77(15):3965–81. doi: 10.1158/0008-5472.CAN-16-2634 PMC8330958 28701486

[29] Qian X Zhao J Yeung PY Zhang QC Kwok CK. Revealing lncRNA structures and interactions by sequ-encing-based approaches. Trends Biochem Sci (2019) 44(1):33–52. doi: 10.1016/j.tibs.2018.09.012 30459069

[30] Chen X Jiang C Qin B Liu G Ji J Sun X. LncRNA ZNF503-AS1 promotes RPE differentiation by down regulating ZNF503 expression. Cell Death Dis (2017) 8(9):e3046. doi: 10.1038/cddis.2017.382 28880276 PMC5636965

[31] Jathar S Kumar V Srivastava J Tripathi V. Technological developments in lncRNA biology. Adv Exp Med Biol (2017) 1008:283–323. doi: 10.1007/978-981-10-5203-3_10 28815544

[32] Schmitz SU Grote P Herrmann BG. Mechanisms of long noncoding RNA function in development and disease. Cell Mol Life Sci (2016) 73(13):2491–509. doi: 10.1007/s00018-016-2174-5 PMC4894931 27007508

[33] Li D Yang C Yin C Zhao F Chen Z Tian Y. LncRNA, important player in bone development and disease. Endocr Metab Immune Disord Drug Targets (2020) 20(1):50–66. doi: 10.2174/1871530319666190904161707 31483238

[34] Zhang X Liang H Kourkoumelis N Wu Z Li G Shang X. Comprehensive analysis of lncRNA and miRNA expression profiles and ceRNA network construction in osteoporosis. Calcif Tissue Int (2020) 106(4):343–54. doi: 10.1007/s00223-019-00643-9 31858161

[35] Li Z Tan H Zhao W Xu Y Zhang Z Wang M. Integrative analysis of DNA methylation and gene expression profiles identifies MIR4435-2HG as an oncogenic lncRNA for glioma progression. Gene (2019) 715:144012. doi: 10.1016/j.gene.2019.144012 31357021

[36] O'Connell TM Markunas CA. DNA methylation and microRNA-based biomarkers for risk of type 2 diabetes. Curr Diabetes Rev (2016) 12(1):20–9. doi: 10.2174/1573399811666150515125557 25981498

[37] Fan L Lei H Zhang S Peng Y Fu C Shu G. Non-canonical signaling pathway of SNAI2 induces EMT in ovarian cancer cells by suppressing miR-222-3p transcription and upregulating PDCD10. Theranostics (2020) 10(13):5895–913. doi: 10.7150/thno.43198 PMC7254989 32483426

[38] Chen L Heikkinen L Wang C Yang Y Sun H Wong G. Trends in the development of miRNA bioinformatics tools. Brief Bioinform (2019) 20(5):1836–52. doi: 10.1093/bib/bby054 PMC7414524 29982332

[39] Liu K Zhao D Wang D. LINC00528 regulates myocardial infarction by targeting the miR-143-3p /COX-2 axis. Bioengineered (2020) 11(1):11–8. doi: 10.1080/21655979.2019.1704535 PMC6961595 31833800

[40] Smirnova L Gräfe A Seiler A Schumacher S Nitsch R Wulczyn FG. Regulation of miRNA expression during neural cellspecification. Eur J Neurosci (2005) 21(6):1469–77. doi: 10.1111/j.1460-9568.2005.03978.x 15845075

[41] Mahmood M Li Z Casciano D Khodakovskaya MV Chen T Karmakar A. Nanostructural materials increase mineralization in bone cells and affect gene expression through miRNA regulation. J Cell Mol Med (2011) 15(11):2297–306. doi: 10.1111/j.1582-4934.2010.01234.x PMC3822941 21143388

[42] Laxman N Rubin CJ Mallmin H Nilsson O Pastinen T Grundberg E. Global miRNA expression and correlation with mRNA levels in primary human bone cells. RNA (2015) 21(8):1433–43. doi: 10.1261/rna.049148.114 PMC4509933 26078267

[43] Wang H Wei P Zhang Y Li Y Yin L. LncRNA TCONS_00023297 regulates the balance of osteogenic and adipogenic differentiation in bone marrow mesenchymal stem cells and the coupling process of osteogenesis and angiogenesis. Front Cell Dev Biol (2021) 9:697858. doi: 10.3389/fcell.2021.697858 34262909 PMC8274487

[44] Zhang Y Zhang N Wei Q Dong Y Liu Y Yuan Q. MiRNA-320a-5p contributes to the homeostasis of osteogenesis and adipogenesis in bone marrow mesenchymal stem cell. Regener Ther (2022) 20:32–40. doi: 10.1016/j.reth.2022.03.001 PMC8968203 35402661

[45] Peng S Cao L He S Zhong Y Ma H Zhang Y. An overview of long noncoding RNAs involved in bone regeneration from mesenchymal stem cells. Stem Cells Int (2018) 2018:8273648. doi: 10.1155/2018/8273648 29535782 PMC5829309

[46] Salmena L Poliseno L Tay Y Kats L Pandolfi PP. A ceRNA hypothesis: the Rosetta Stone of a hidden RNA language? Cell (2011) 146(3):353–8. doi: 10.1016/j.cell.2011.07.014 PMC3235919 21802130

[47] Zhao Y Ma S Cui Z Li S Chen Y Yin Y. The relationship between LncRNAs and lung adenocarcinoma as well as their ceRNA network. Cancer biomark (2021) 31(2):165–76. doi: 10.3233/CBM-203078 PMC12500015 33896828

[48] Xu Y Wang S Tang C Chen W. Upregulation of long non-coding RNA HIF 1α-anti-sense 1 induced by transforming growth factor-β-mediated targeting of sirtuin1 promotes osteoblastic differentiation of human bone marrow stromal cells. Mol Med Rep (2015) 12(5):7233–8. doi: 10.3892/mmr.2015.4415 PMC4626181 26460121

[49] Paraskevopoulou MD Hatzigeorgiou AG. Analyzing miRNA-lncRNA interactions. Methods Mol Biol (2016) 1402:271–86. doi: 10.1007/978-1-4939-3378-5_21 26721498

[50] Sen R Ghosal S Das S Balti S Chakrabarti J. Competing endogenous RNA: the key to posttranscriptional regulation. Sci World J (2014) 2014:896206. doi: 10.1155/2014/896206 PMC3929601 24672386

[51] Wu Y Xie L Wang M Xiong Q Guo Y Liang Y. Mettl3-mediated m^6^A RNA methylation regulates the fate of bone marrow mesenchymal stem cells and osteoporosis. Nat Commun (2018) 9(1):4772. doi: 10.1038/s41467-018-06898-4 30429466 PMC6235890

[52] Arthur A Zannettino A Gronthos S. The therapeutic applications of multipotential mesenchymal/stromal stem cells in skeletal tissue repair. J Cell Physiol (2009) 218(2):237–45. doi: 10.1002/jcp.21592 18792913

[53] Li X Guo L Liu Y Su Y Xie Y Du J. MicroRNA-21 promotes osteogenesis of bone marrow mesenchymal stem cells via the Smad7-Smad1/5/8-Runx2 pathway. Biochem Biophys Res Commun (2017) 493(2):928–33. doi: 10.1016/j.bbrc.2017.09.119 28943430

[54] Wang X Chen T Deng Z Gao W Liang T Qiu X. Melatonin promotes bone marrow mesenchymal stem cell osteogenic differentiation and prevents osteoporosis development through modulating circ_0003865 that sponges miR-3653-3p. Stem Cell Res Ther (2021) 12(1):150. doi: 10.1186/s13287-021-02224-w 33632317 PMC7908669

[55] Zhou Q An Y Tang Y. Long noncoding RNA-regulator of reprogramming alleviates hypoxia -induced cerebral injury in mice model and human via modulating apoptosis complexes. J Integr Neurosci (2019) 18(4):431–7. doi: 10.31083/j.jin.2019.04.1194 31912702

[56] Mevel R Draper JE Lie-A-Ling M Kouskoff V Lacaud G. RUNX transcription factors: orchestrators of development. Development (2019) 146(17):dev148296. doi: 10.1242/dev.148296 31488508

[57] Huang J Zhao L Xing L Chen D. MicroRNA-204 regulates Runx2 protein expression and mesenchymal progenitor cell differentiation. Stem Cells (2010) 28(2):357–64. doi: 10.1002/stem.288 PMC2837600 20039258

[58] Jiang Y Zhang J Li Z Jia G. Bone marrow mesenchymal stem cell-derived exosomal miR-25 regulates the ubiquitination and degradation of runx2 by SMURF1 to promote fracture healing in mice. Front Med (Lausanne) (2020) 7:577578. doi: 10.3389/fmed.2020.577578 33425934 PMC7793965

[59] Ouyang Z Kang D Li K Liang G Liu Z Mai Q. DEPTOR exacerbates bone-fat imbalance in osteoporosis by transcriptional-ly modulating BMSC differentiation. BioMed Pharmacother (2022) 151:113164. doi: 10.1016/j.biopha.2022.113164 35609371

[60] Komori T. Roles of runx2 in skeletal development. Adv Exp Med Biol (2017) 962:83–93. doi: 10.1007/978-981-10-3233-2_6 28299652

[61] Zhang HL Du XY Dong QR. LncRNA XIXT promotes osteogenic differentiation of bone mesenchymal stem cells and alleviates osteoporosis progression by targeting miRNA-30a-5p. Eur Rev Med Pharmacol Sci (2019) 23(20):8721–9. doi: 10.26355/eurrev_201910_19266 31696458

[62] Gao GC Yang DW Liu W. LncRNA TERC alleviates the progression of osteoporosis by absorbing miRNA-217 to upregulate RUNX2. Eur Rev Med Pharmacol Sci (2020) 24(2):526–34. doi: 10.26355/eurrev_202001_20029 32016954

[63] Feng J Wang JX Li CH. LncRNA GAS5 overexpression alleviates the development ofosteoporosis through promoting osteogenic differentiation of MSCs via targeting micro-RNA-498 to regulate RUNX2. Eur Rev Med Pharmacol Sci (2019) 23(18):7757–65. doi: 10.26355/eurrev_201909_18985 31599401

[64] Yu H Li Y Tang J Lu X Hu W Cheng L. Long non-coding RNA RP11-84C13.1 promotes osteogenic differentiation of bone mesenchymal stem cells and alleviates osteopor-osis progression via the miR-23b-3p/RUNX2 axis. Exp Ther Med (2021) 22(5):1340. doi: 10.3892/etm.2021.10775 34630694 PMC8495569

[65] Soung do Y Dong Y Wang Y Zuscik MJ Schwarz EM O'Keefe RJ. Runx3/AML2/Cbfa3 regulates early and late chondrocyte differentiation. J Bone Miner Res (2007) 22(8):1260–70. doi: 10.1359/jbmr.070502 17488194

[66] Zhang J Xu N Yu C Miao K Wang Q. LncRNA PART1/miR-185-5p/RUNX3 feedback loop modulates osteogenic differentiation of bone marrow mesenchymal stem cells. Autoimmunity (2021) 54(7):422–9. doi: 10.1080/08916934.2021.1966771 34431433

[67] Zheng J Guo H Qin Y Liu Z Ding Z Zhang L. SNHG5/miR-582-5p/RUNX3 feedback loop regulates osteogenic differentiation and apoptosis of bone marrow mesenchymal stem cells. J Cell Physiol (2020) 1–12. doi: 10.1002/jcp.29527 33111341

[68] Chen F Bi D Cao G Cheng C Ma S Liu Y. Bone morphogenetic protein 7-transduced human dermal-derived fibroblast cells differentiate into osteoblasts and form bone. vivo. Connect Tissue Res (2018) 59(3):223–32. doi: 10.1080/03008207.2017.1353085 28696808

[69] Gu H Song M Boonanantanasarn K Baek K Woo KM Ryoo HM. Conditions inducing excessive O-glcNAcylation inhibit BMP2-induced osteogenic differentiation of C2C12 cells. Int J Mol Sci (2018) 19(1):202. doi: 10.3390/ijms19010202 29315243 PMC5796151

[70] Valadares ER Carneiro TB Santos PM Oliveira AC Zabel B. What is new in genetics and osteogenesis imperfecta classification? J Pediatr (Rio J) (2014) 90(6):536–41. doi: 10.1016/j.jped.2014.05.003 25046257

[71] Zhang Y Chen B Li D Zhou X Chen Z. LncRNA NEAT1/miR-29b-3p/BMP1 axis promotes osteogenic differentiation in human bone marrow-derived mesenchymal stem cells. Pathol Res Pract (2019) 215(3):525–31. doi: 10.1016/j.prp.2018.12.034 30638953

[72] Zhang N Hu X He S Ding W Wang F Zhao Y. LncRNA MSC-AS1 promotes osteogenic differentiation anda-lleviates osteoporosis through sponging microRNA-140-5p to upregulate BMP2. Biochem Biophys Res Commun (2019) 519(4):790–6. doi: 10.1016/j.bbrc.2019.09.058 31551149

[73] Wang CG Liao Z Xiao H Liu H Hu YH Liao QD. LncRNA KCNQ1OT1 promoted BMP2 expression to regulate osteogenic differentiation by sponging miRNA-214. Exp Mol Pathol (2019) 107:77–84. doi: 10.1016/j.yexmp.2019.01.012 30703347

[74] Zhao Y Chen Y Hu X Zhang N Wang F. lncRNA LINC01535 upregulates BMP2 expression levels to promote osteogenic differentiation via sponging miR-3619-5p. Mol Med Rep (2020) 22(6):5428–35. doi: 10.3892/mmr.2020.11635 33174047

[75] Asila A Yang X Kaisaer Y Ma L. SNHG16/miR-485-5p/BMP7 axis modulates osteogenicdifferentiation of human bone marrow-derived mesenchymal stem cells. J Gene Med (2021) 23(3):e3296. doi: 10.1002/jgm.3296 33179372

[76] Garcia J Delany AM. MicroRNAs regulating TGFβ and BMP signaling in the osteoblast lineage. Bone (2021) 143:115791. doi: 10.1016/j.bone.2020.115791 33285257 PMC7787082

[77] Martinez-Hackert E Sundan A Holien T. Receptor binding competition: A paradigm for regulating TGF-β family action. Cytokine Growth Factor Rev (2021) 57:39–54. doi: 10.1016/j.cytogfr.2020.09.003 33087301 PMC7897244

[78] Jia S Meng A. TGF-β family signaling and development. Development (2021) 148(5):dev188490. doi: 10.1242/dev.188490 33712443

[79] Tzavlaki K Moustakas A. TGF-β Signaling. Biomolecules (2020) 10(3):487. doi: 10.3390/biom10030487 32210029 PMC7175140

[80] Han Y Yang Q Huang Y Jia L Zheng Y Li W. Long non-coding RNA SNHG5 prom-otes the osteogenic differentiation of bone marrow mesenchymal stem cells via the miR-212-3p/GDF5/SMAD pathway. Stem Cell Res Ther (2022) 13(1):130. doi: 10.1186/s13287-022-02781-8 35346361 PMC8962127

[81] Wang JL Wei X Wang AG Bai Y Wu XJ. KCNQ1OT1 regulates osteogenic differentiation of hBMSC by miR-320a/Smad5 axis. Eur Rev Med Pharmacol Sci (2020) 24(6):2843–54. doi: 10.26355/eurrev_202003_20648 32271402

[82] Cao L Liu W Zhong Y Zhang Y Gao D He T. Linc02349 promotes osteogenesis of human umbilical cord-derived stem cells by acting as a competing endogenous RNA for miR-25-3p and miR-33b-5p. Cell Prolif (2020) 53(5):e12814. doi: 10.1111/cpr.12814 32346990 PMC7260076

[83] Wu D Yin L Sun D Wang F Wu Q Xu Q. Long noncoding RNA TUG1 promotes osteogenic differentiation of human periodontal ligament stem cell through sponging microRNA-222-3p to negativelyregulate Smad2/7. Arch Oral Biol (2020) 117:104814. doi: 10.1016/j.archoralbio.2020.104814 32574885

[84] Wei B Wei W Zhao B Guo X Liu S. Long non-coding RNA HOTAIR inhibits miR-17-5p to regulate osteogenic differentiation and proliferation in non-traumatic osteonecrosis of femoral head. PloS One (2017) 12(2):e0169097. doi: 10.1371/journal.pone.0169097 28207735 PMC5312925

[85] Zang LY Yang XL Li WJ Liu GL. Long noncoding RNA metastasis-associated lung adenocarcinoma transcript 1 promotes the osteoblast differentiation of human bone marrow-derived mesenchymal stem cells by targeting the microRNA-96/osterix axis. J Craniofac Surg (2022) 33(3):956–61. doi: 10.1097/SCS.0000000000008092 34456284

[86] Gao Y Xiao F Wang C Wang C Cui P Zhang X. Long noncoding RNA MALAT1 promotes osterix express-ion to regulate osteogenic differentiation by targeting miRNA-143 in human bone marrowderived mesenchymal stem cells. J Cell Biochem (2018) 119(8):6986–96. doi: 10.1002/jcb.26907 29741283

[87] Liu ZH Qi DD Li X Zhang SQ Zhao Y Fu LX. LncRNA SNHG14 promotes osteogenic differentiation of human bone marrow-derived mesenchymal stem cells via osteogenic regulating miR-185-5p/WISP2 axis. J Biol Regul Homeost Agents (2021) 35(2):605–15. doi: 10.23812/20-391-A 33928771

[88] Du M Wu B Fan S Liu Y Ma X Fu X. SNHG14 induces osteogenic differentiation of human stromal (mesenchymal) stem cells in *vitro* by downregulating miR-2861. BMC Musculoskelet Disord (2020) 21(1):525. doi: 10.1186/s12891-020-03506-9 32770994 PMC7415173

[89] Xu Y Xin R Sun H Long D Li Z Liao H. Long Non-coding RNAs LOC100126784 and POM121L9P Derived From Bone Marrow Mesenchymal Stem Cells Enhance Osteogenic Differentiation via the miR-503-5p/SORBS1 Axis. Front Cell Dev Biol (2021) 9:723759. doi: 10.3389/fcell.2021.723759 34746123 PMC8570085

[90] Yang X Yang J Lei P Wen T. LncRNA MALAT1 shuttled by bone marrow-derived mesenchymal stem cells-secreted exosomes alleviates osteoporosis through mediating microRNA-34c/SATB2 axis. Aging (Albany NY) (2019) 11(20):8777–91. doi: 10.18632/aging.102264 PMC6834402 31659145

[91] Tang JZ Zhao GY Zhao JZ Di DH Wang B. lncRNA IGF2-AS promotes the osteogenic differentiation of bone marrow mesenchymal stem cells by sponging miR-3,126-5p to upregulate KLK4. J Gene Med (2021) 23(10):e3372. doi: 10.1002/jgm.3372 34101307

[92] Yang JJ Peng WX Zhang MB. LncRNA KCNQ1OT1 promotes osteogenic differe-ntiation via miR-205-5p/RICTOR axis. Exp Cell Res (2022) 415(1):113119. doi: 10.1016/j.yexcr.2022.113119 35341776

[93] Wang X Zhao D Zhu Y Dong Y Liu Y. Long non-coding RNA GAS5 promotes osteogenic differentiation of bone marrow mesenchymal stem cells by regulating the miR-135a-5p/FOXO1 pathway. Mol Cell Endocrinol (2019) 496:110534. doi: 10.1016/j.mce.2019.110534 31398367

[94] Li X. LncRNA metastasis-associated lung adenocarcinoma transcript-1 promotes osteogenic differentiation of bone marrow stem cells and inhibits osteoclastic differentiation of Mø in osteoporosis via the miR-124-3p/IGF2BP1/Wnt/β-catenin axis. J Tissue Eng Regener Med (2022) 16(3):311–29. doi: 10.1002/term.3279 34962086

[95] Ren L Guo L Kou N Lv J Wang Z Yang K. LncRNA LINC00963 promotes osteogenic differentiation of hBMSCs and alleviates osteoporosis progression by targeting miRNA-760/ETS1 axis. Autoimmunity (2021) 54(6):313–25. doi: 10.1080/08916934.2021.1922890 34184952

[96] Shi ZL Zhang H Fan ZY Ma W Song YZ Li M. Long noncoding RNA LINC00314 facilitates osteogenic differentiation of adipose-derived stem cells through the hsa-miR-129-5p/GRM5 axis via the Wnt signaling pathway. Stem Cell Res Ther (2020) 11(1):240. doi: 10.1186/s13287-020-01754-z 32552820 PMC7302136

[97] Shen G Ren H Shang Q Zhao W Zhang Z Yu X. Foxf1 knockdown promotes BMSC osteogenesis in part by activating the Wnt/β-catenin signalling pathway and prevents ovariectomy-induced bone loss. EBio Med (2020) 52:102626. doi: 10.1016/j.ebiom.2020.102626 PMC6992955 31981979

[98] Zhang Y Zhou L Fu Q Liu Z. ANKRD1 activates the Wnt signaling pathway by modulating CAV3 expression and thus promotes BMSC osteogenic differentiation and bone formation in ovariectomized mice. Biochim Biophys Acta Mol Basis Dis (2023) 1869(5):166693. doi: 10.1016/j.bbadis.2023.166693 36958710

[99] Liang WC Fu WM Wang YB Sun YX Xu LL Wong CW. H19 activates Wnt signaling and promotes osteoblast differentiation by functioning as a competing endogenous RNA. Sci Rep (2016) 6:20121. doi: 10.1038/srep20121 26853553 PMC4745008

[100] Feng L Shi L Lu YF Wang B Tang T Fu WM. Linc-ROR Promotes Osteogenic Differentiation of Mesenchymal Stem Cells by Functioning as a Competing Endogenous RNA for miR-138 and miR-145. Mol Ther Nucleic Acids (2018) 11:345–53. doi: 10.1016/j.omtn.2018.03.004 PMC5992460 29858070

[101] Cai WL Zeng W Liu HH Zhu BY Liu JL Liu Y. LncRNALINC00707 promotes osteogenic dif ferentiation of hBMSCs through the Wnt/β-catening pathway activated by LINC00707/mi R-145/LRP5 axis. Eur Rev Med Pharmacol Sci (2020) 24(1):18–28. doi: 10.26355/eurrev_202001_19891 31957814

[102] Jia B Wang Z Sun X Chen J Zhao J Qiu X. Long noncoding RNA LINC00707 sponges miR-370-3p to promote osteogenesis of human bone marrow-derived mesenchymalstem cells through upregulating WNT2B. Stem Cell Res Ther (2019) 10(1):67. doi: 10.1186/s13287-019-1161-9 30795799 PMC6387535

[103] Jullien N Maudinet A Leloutre B Ringe J Häupl T Marie PJ. Downregulation of ErbB3 by Wnt3a contributes to wnt-induced osteoblast differentiation in mesenchymal cells. J CellBiochem (2012) 113(6):2047–56. doi: 10.1002/jcb.24076 22274864

[104] Felber K Elks PM Lecca M Roehl HH. Expression of osterix Is Regulated by FGF and Wnt/β-Catenin Signalling during Osteoblast Differentiation. PloS One (2015) 10(12):e0144982. doi: 10.1371/journal.pone.0144982 26689368 PMC4686927

[105] Chen S Li Y Zhi S Ding Z Huang Y Wang W. lncRNA xist regulates osteoblast differentiation by sponging miR-19a-3p in aging-induced osteoporosis. Aging Dis (2019) 11(5):1058–68. doi: 10.14336/AD.2019.0724 PMC7505278 33014522

[106] Yu J Xiao M Ren G. Long non-coding RNA XIST promotes osteoporosis by inhibiting the differentiation of bone marrow mesenchymal stem cell by sponging miR-29b-3p that suppresses nicotinamide N-methyltransferase. Bioengineered (2021) 12(1):6057–69. doi: 10.1080/21655979.2021.1967711 PMC8806730 34486487

[107] Wang W Li T Feng S. Knockdown of long non-coding RNA HOTAIR promotes bone marrow mesenchymal stem cell differentiation by sponging microRNA miR-378g that inhibits nicotinamide N-methyltransferase. Bioengineered (2021) 12(2):12482–97. doi: 10.1080/21655979.2021.2006863 PMC8810179 34895051

[108] Weng W Di S Xing S Sun Z Shen Z Dou X. Long non-coding RNA DANCR modulates osteogenic differentiation by regulating the miR-1301-3p/PROX1 axis. Mol Cell Biochem (2021) 476(6):2503–12. doi: 10.1007/s11010-021-04074-9 33629241

[109] Xiang J Fu HQ Xu Z Fan WJ Liu F Chen B. lncRNA SNHG1 attenuates osteogenic differentiation via the miR-101/DKK1 axis in bone marrow mesenchymal stem cells. Mol Med Rep (2020) 22(5):3715–22. doi: 10.3892/mmr.2020.11489 PMC7533455 32901867

[110] Che M Gong W Zhao Y Liu M. Long noncoding RNA HCG18 inhibits the differentiation of human bone marrow-derived mesenchymal stem cells in osteoporosis by targeting miR-30a-5p/NOTCH1 axis. Mol Med (2020) 26(1):106. doi: 10.1186/s10020-020-00219-6 33176682 PMC7656763

[111] Wang F Deng H Chen J Wang Z Yin R. LncRNA MIAT can regulate the proli-feration, apoptosis,and osteogenic differentiation of bone marrow-derived mesenchymal stem cells by targeting miR-150-5p. Bioengineered (2022) 13(3):6343–52. doi: 10.1080/21655979.2021.2011632 PMC9208443 35282774

[112] Lu F Tang L. LncRNA BC083743 silencing exacerbated osteoporosis by regulating the miR-103-3p/SATB2 axis to inhibit osteogenic differentiation. Comput Intell Neurosci (2022) 2022:7066759. doi: 10.1155/2022/7066759 35769281 PMC9236849

[113] Wang Q Li Y Zhang Y Ma L Lin L Meng J. LncRNA MEG3 inhibited osteogenic differentiation of bone marrow mesenchymal stem cells from postmenopausal osteoporosis by targeting miR-133a-3p. BioMed Pharmacother (2017) 89:1178–86. doi: 10.1155/2022/7066759 28320084

[114] Li M Cong R Yang L Yang L Zhang Y Fu Q. A novel lncRNA LNC_000052 leads to the dysfunction ofosteoporotic BMSCs via the miR-96-5p-PIK3R1 axis. Cell Death Dis (2020) 11(9):795. doi: 10.1038/s41419-020-03006-7 32968049 PMC7511361

[115] Xiaoling G Shuaibin L Kailu L. MicroRNA-19b-3p promotes cell proliferation and osteogenic differentiation of BMSCs by interacting with lncRNA H19. BMC Med Genet (2020) 21(1):11. doi: 10.1186/s12881-020-0948-y 31918667 PMC6953218

[116] Vazquez-Villegas ML Rodriguez-Jimenez NA Contreras-Haro B Vasquez-Jimenez JC Perez-Guerrero EE Moran-Moguel MC. Genotypic Analyses of the Sclerostin rs851056 and Dickkopf rs1569198 Polymorphisms in Mexican-Mestizo Postmenopausal Osteopor-osis: A Case-Control Study. Genet Test Mol Biomarkers (2021) 25(3):211–7. doi: 10.1089/gtmb.2020.0199 33734895

[117] Holdsworth G Greenslade K Jose J Stencel Z Kirby H Moore A. Dampening of the bone formation response following repeat dosing with sclerostin antibody in mice is associated with up-regulation of Wnt antagonists. Bone (2018) 107:93–103. doi: 10.1016/j.bone.2017.11.003 29129759

[118] Yu X Rong PZ Song MS Shi ZW Feng G Chen XJ. lncRNA SNHG1 induced by SP1 regulates bone remodeling and angiogenesis via sponging miR-181c-5p and modulating SFRP1/Wnt signaling pathway. Mol Med (2021) 27(1):141. doi: 10.1186/s10020-021-00392-2 34732133 PMC8565074

[119] Wang CG Hu YH Su SL Zhong D. LncRNA DANCR and miR-320a suppressed osteogenic differentiation in osteoporosis by directly inhibiting the Wnt/β-catenin signaling pathway. Exp Mol Med (2020) 52(8):1310–25. doi: 10.1038/s12276-020-0475-0 PMC8080634 32778797

[120] Ren M Liu H Jiang W Zhou Z Yao X Liu Z. Melatonin repairs osteoporotic bone defects in iron-overloaded rats through PI3K/AKT/GSK-3*β*/P70S6k signaling pathway. Oxid Med Cell Longev (2023) 2023:7718155. doi: 10.1155/2023/7718155 36703914 PMC9873465

[121] Zhang B Yang LL Ding SQ Liu JJ Dong YH Li YT. Anti-osteoporotic activity of an edible traditional chinese medicine cistanche deserticola on bone metabolism of ovariectomized rats through RANKL/RANK/TRAF6-mediated signaling pathways. Front Pharmacol (2019) 10:1412. doi: 10.3389/fphar.2019.01412 31849666 PMC6902040

[122] Zhang X Cui J Cheng L Lin K. Enhancement of osteoporotic bone regeneration by strontium-substituted 45S5 bioglass via time-dependent modulation of autophagy and the Akt/mTOR signaling pathway. J Mater Chem B (2021) 9(16):3489–501. doi: 10.1039/d0tb02991b 33690737

[123] Wang Y Luo TB Liu L Cui ZQ. LncRNA LINC00311 promotes the proliferation and differentiation of osteoclasts in osteoporotic rats through the notch signaling pathway by targeting DLL3. Cell Physiol Biochem (2018) 47(6):2291–306. doi: 10.1159/000491539 29975944

[124] Lu W Zheng C Zhang H Cheng P Miao S Wang H. Hedgehog signaling regulates bone homeostasis through orchestrating osteoclast differentiation and osteoclast-osteoblast coupling. Cell Mol Life Sci (2023) 80(6):171. doi: 10.1007/s00018-023-04821-9 37261512 PMC11071711

[125] Zhong LN Zhang YZ Li H Fu HL Lv CX Jia XJ. Overexpressed miR-196a accelerates osteogenic differentiation in osteoporotic mice via GNAS-dependent Hedgehog signaling pathway. J Cell Biochem (2019) 120(12):19422–31. doi: 10.1002/jcb.29166 31452264

[126] Ragni E Colombini A De Luca P Libonati F Viganò M Perucca Orfei C. miR-103a-3p and miR-22-5p are reliableReference genes in extracellular vesicles from cartilage, adipose tissue, and bone marrow cells. Front Bioeng Biotechnol (2021) 9:632440. doi: 10.3389/fbioe.2021.632440 33659243 PMC7917212

[127] Discher DE Mooney DJ Zandstra PW. Growth factors, matrices, and forces combine and control stem cells. Science (2009) 324(5935):1673–7. doi: 10.1126/science.1171643 PMC2847855 19556500

[128] Tsai MC Manor O Wan Y Mosammaparast N Wang JK Lan F. Long noncoding RNA as modular scaffold of hist-one modification complexes. Science (2010) 329(5992):689–93. doi: 10.1126/science.1192002 PMC2967777 20616235

[129] Yang Y Yujiao W Fang W Linhui Y Ziqi G Zhichen W. The roles of miRNA, lncRNA and circRNA in the development of osteoporosis. Biol Res (2020) 53(1):40. doi: 10.1186/s40659-020-00309-z 32938500 PMC7493179

[130] Yin C Tian Y Li D Yu Y Jiang S Hou Y. Long noncoding RNA Lnc-DIF inhibits bone formation by sequestering miR-489-3p. I Sci (2022) 25(3):103949. doi: 10.1016/j.isci.2022.103949 PMC8898894 35265818

[131] Zhang L Xie H Li S. LncRNA LOXL1-AS1 controls osteogenic and adipocytic differentiation of bone marrow mesenchymal stem cells in postmenopausal osteoporosis through regulating the miR-196a-5p/Hmga2 axis. J Bone Miner Metab (2020) 38(6):794–805. doi: 10.1007/s00774-020-01123-z 32651705

[132] Shi Z Zhong Q Chen Y Luo X. Long noncoding RNA ZBTB40-IT1 regulates bone mass by directing the differentiation of human bone marrow mesenchymal stromal cells via the microRNA-514a-3p/FOXO4 axis. Hum Cell (2022) 35(5):1408–23. doi: 10.1007/s13577-022-00730-4 35676609

[133] Yan S Tang Z Chen K Liu Y Yu G Chen Q. Long noncoding RNA MIR31HG inhibits hepatocellular carcinoma proliferation and metastasis by sponging microRNA-575 to modulate ST7L expression. J Exp Clin Cancer Res (2018) 37(1):214. doi: 10.1186/s13046-018-0853-9 30176933 PMC6122648

[134] Li M Xie Z Wang P Li J Liu W Tang S. The long noncoding RNA GAS5 negatively regulates the adipogenic differentiation of MSCs by modulating the Luby AO /CTGF axis as a ceRNA. Cell Death Dis (2018) 9(5):554. doi: 10.1038/s41419-018-0627-5 29748618 PMC5945827

[135] Letarouilly JG Broux O Clabaut A. New insights into the epigenetics of osteoporosis. Genomics (2018) 111(4):793–8. doi: 10.1016/j.ygeno.2018.05.001 29730394

[136] Słupski W Jawień P Nowak B. Botanicals in postmenopausal osteoporosis. Nutrients (2021) 13(5):1609. doi: 10.3390/nu13051609 34064936 PMC8151026

[137] Xu SY Shi P Zhou RM. Post-menopausal oestrogen deficiency induces osteoblast apoptosis via regulating HOTAIR/miRNA-138 signalling and suppressing TIMP1 expression. J Cell Mol Med (2021) 25(10):4572–82. doi: 10.1111/jcmm.16216 PMC8107111 33733597

[138] Chai C Hong F Yan Y Yang L Zong H Wang C. Effect of traditional Chinese medicine formula GeGen decoction on primary dysmenorrhea: A randomized controlled trial study. J Ethnopharmacol (2020) 261:113053. doi: 10.1016/j.jep.2020.113053 32534120

[139] Zhang C Sui X Jiang Y Wang X Wang S. Antitumor effects of icaritin and the molecular mechanisms. Discovery Med (2020) 29(156):5–16. 32598859

[140] Zhao P Soukup ST Hegevoss J Ngueu S Kulling SE Diel P. Anabolic effect of the traditional Chinese medicine compound tanshinone IIA on myotube hypertrophy is mediated by estrogen receptor. Planta Med (2015) 81(7):578–85. doi: 10.1055/s-0035-1545883 26018796

[141] Zhao Y Shao G Liu X Li Z. Assessment of the therapeutic potential of melatonin for the treatment of osteoporosis through a narrative review of its signaling and preclinical and clinical studies. Front Pharmacol (2022) 13:866625. doi: 10.3389/fphar.2022.866625 35645810 PMC9130700

[142] Claustrat B Leston J. Melatonin: Physiological effects in humans. Neurochirur-gie (2015) 61(2-3):77–84. doi: 10.1016/j.neuchi.2015.03.002 25908646

[143] Vasey C McBride J Penta K. Circadian rhythm dysregulation and restoration: the role of melatonin. Nutrients (2021) 13(10):3480. doi: 10.3390/nu13103480 34684482 PMC8538349

[144] Reiter RJ Rosales-Corral S Tan DX Jou MJ Galano A Xu B. Melatonin as a mitochondria-targeted antioxidant: one of evolution's best ideas. Cell Mol Life Sci (2017) 74(21):3863–81. doi: 10.1007/s00018-017-2609-7 PMC11107735 28864909

[145] Day D Burgess CM Kircik LH. Assessing the potential role for topical melatonin in an antiaging skin regimen. J Drugs Dermatol (2018) 17(9):966–9. 30235383

[146] Luo J Zhang Z Sun H Song J Chen X Huang J. Effect of melatonin on T/B cell activation and immune regulation in pinealectomy mice. Life Sci (2020) 242:117191. doi: 10.1016/j.lfs.2019.117191 31863775

[147] Li T Jiang S Lu C Yang W Yang Z Hu W. Melatonin: Another avenue for treating osteoporosis? J Pineal Res (2019) 66(2):e12548. doi: 10.1111/jpi.12548 30597617

[148] Wang B Wen H Smith W Hao D He B Kong L. Regulation effects of melatonin on bone marrow mesenchymal stem cell differentiation. J Cell Physiol (2019) 234(2):1008–15. doi: 10.1002/jcp.27090 30145787

[149] Han H Tian T Huang G Li D Yang S. The lncRNA H19/miR-541-3p/Wnt/β-catenin axis plays a vital role in melatonin-mediated osteogenic differentiation of bone marrow mesenchymal stemcells. Aging (Albany NY) (2021) 13(14):18257–73. doi: 10.18632/aging.203267 PMC8351702 34311444

[150] Wang W Mao J Chen Y Zuo J Chen L Li Y. Naringin promotes osteogenesis and ameliorates osteoporosis development by targeting JAK2/STAT3 signalling. Clin Exp Pharmacol Physiol (2022) 49(1):113–21. doi: 10.1111/1440-1681.13591 34525226

[151] Yang Z Huang JH Liu SF Zhao YJ Shen ZY Wang YJ. The osteoprotective effect of psoralen in ovariectomy-induced osteoporotic rats via stimulating the osteoblastic differentiation from bone mesenchymal stem cells. Menopause (2012) 19(10):1156–64. doi: 10.1097/gme.0b013e3182507e18 22781784

[152] Cole IB Cao J Alan AR Saxena PK Murch SJ. Comparisons of Scutellaria baicalensis, Scutellaria lateriflora and Scutellaria racemosa: genome size, antioxidant potential and phytochemistry. Planta Med (2008) 74(4):474–81. doi: 10.1055/s-2008-1034358 18484546

[153] Pang XG Cong Y Bao NR Li YG Zhao JN. Quercetin stimulates bone marrow mesenchymal stem cell differentiation through an estrogen receptor-mediated pathway. BioMed Res Int (2018) 2018:4178021. doi: 10.1155/2018/4178021 29736392 PMC5875037

[154] Wang N Wang L Yang J Wang Z Cheng L. Quercetin promotes osteogenic differentiation and antioxidant responses of mouse bone mesenchymal stem cells through activation of the AMPK/SIRT1 signaling pathway. Phytother Res (2021) 35:2639–50. doi: 10.1002/ptr.7010 33421256

[155] Bian W Xiao S Yang L Chen J Deng S. Quercetin promotes bone marrow mesenchymal stem cell proliferation and osteogenic differentiation through the H19/miR-625-5p axis to activate the Wnt/β-catenin pathway. BMC Complement Med Ther (2021) 21(1):243. doi: 10.1186/s12906-021-03418-8 34592982 PMC8485455

[156] Britton RG Kovoor C Brown K. Direct molecular targets of resveratrol: identifying key interactions to unlock complex mechanisms. Ann N Y Acad Sci (2015) 1348(1):124–33. doi: 10.1111/nyas.12796 26099829

[157] Csiszar A. Anti-inflammatory effects of resveratrol: possible role in prevention of agerelated cardiovascular disease. Ann N Y Acad Sci (2011) 1215:117–22. doi: 10.1111/j.1749-6632.2010.05848.x PMC3058481 21261649

[158] Wang B Wang Y Xu K Zeng Z Xu Z Yue D. Resveratrol alleviates sepsis-induced acute kidney injury by deactivating the lncRN-A MALAT1/MiR-205 axis. Cent Eur J Immunol (2021) 46(3):295–304. doi: 10.5114/ceji.2021.109195 34764801 PMC8574118

[159] Xia D Sui R Zhang Z. Administration of resveratrol improved Parkinson's disease- like phenotype by suppressing apoptosis of neurons via modulating the MALAT1/miR-129/SNCA signaling pathway. J Cell Biochem (2019) 120(4):4942–51. doi: 10.1002/jcb.27769 30260025

[160] Yang K Li W Duan W Jiang Y Huang N Li Y. Resveratrol attenuates pulmonary embolism associated cardiac injury by suppressing activation of the inflammasome via the MALAT1-miR-22-3p signaling pathway. Int J Mol Med (2019) 44(6):2311–20. doi: 10.3892/ijmm.2019.4358 31573048

[161] Peltz L Gomez J Marquez M Alencastro F Atashpanjeh N Quang T. Resveratrol exerts dosage and duration dependent effect on human mesenchymal stem cell development. PloS One (2012) 7(5):e37162. doi: 10.1371/journal.pone.0037162 22615926 PMC3353901

[162] Teng JW Bian SS Kong P Chen YG. Icariin triggers osteogenic differentiation of bone marrow stem cells by up-regulating miR-335-5p. Exp Cell Res (2022) 414(2):113085. doi: 10.1016/j.yexcr.2022.113085 35292240

[163] Gao J Xiang S Wei X Yadav RI Han M Zheng W. Icariin promotes the osteogenesis of bone marrow mesenchymal stem cells through regulating sclerostin and activating the wnt/β-catenin signaling pathway. BioMed Res Int (2021) 2021:6666836. doi: 10.1155/2021/6666836 33553429 PMC7847333

[164] Liu T Chi H Chen J Chen C Huang Y Xi H. Curcumin suppresses proliferation and in *vitro* invasion of human prostate cancer stem cells by ceRNA effect of miR-145 and lncRNA-ROR. Gene (2017) 631:29–38. doi: 10.1016/j.gene.2017.08.008 28843521

[165] Liu JM Li M Luo W Sun HB. Curcumin attenuates Adriamycin-resistance of acute myeloid leukemia by inhibiting the lncRNA HOTAIR/miR-20a-5p/WT1 axis. Lab Invest (2021) 101(10):1308–17. doi: 10.1038/s41374-021-00640-3 34282279

[166] Kim WK Ke K Sul OJ Kim HJ Kim SH Lee MH. Curcumin protects against ovariectomy-induced bone lossand decreases osteoclastogenesis. J Cell Biochem (2011) 112(11):3159–66. doi: 10.1002/jcb.23242 21732406

[167] Son HE Kim EJ Jang WG. Curcumin induces osteoblast differentiation through mild-endoplasmic reticulum stress-mediated such as BMP2 on osteoblast cells. Life Sci (2018) 193:34–9. doi: 10.1016/j.lfs.2017.12.008 29223538

[168] Ouyang X Ding Y Yu L Xin F Yang X. LncRNA TUG regulates osteogenic differentiation of bone marrow mesenchymal stem cells via miRNA-204/SIRT1. J Musculoskelet Neuronal Interact (2022) 22(3):401–10. PMC9438524 36046997

[169] Wu M Dai M Liu X Zeng Q Lu Y. lncRNA SERPINB9P1 Regulates SIRT6 Mediated Osteogenic Differentiation of BMSCs via miR-545-3p. Calcif Tissue Int (2023) 112(1):92–102. doi: 10.1007/s00223-022-01034-3 36348062

[170] Yin J Zheng Z Zeng X Zhao Y Ai Z Yu M. lncRNA MALAT1 mediates osteogenic differentiation ofbone mesenchymal stem cells by sponging miR-129-5p. Peer J (2022) 10:e13355. doi: 10.7717/peerj.13355 35480561 PMC9037136

[171] Shao Y Hu X Wu X. LncRNA X inactive-specific transcript promotes osteoclast differentiation through Tgif2 by acting as a ceRNA of miR-590-3p in a murine model.Regener Med (2021) 16(7):643–53. doi: 10.2217/rme-2020-0174 34187170

[172] Zhang Y Chen XF Li J He F Li X Guo Y. lncRNA neat1 stimulates osteoclastogenesis via sponging miR-7. J Bone Miner Res (2020) 35(9):1772–81. doi: 10.1002/jbmr.4039 32353178

[173] Ling L Hu HL Liu KY Ram YI Gao JL Cao YM. Long noncoding RNA MIRG induces osteoclastogenesis and bone resorption in osteoporosis through negative regulation of miR-1897. Eur Rev Med Pharmacol Sci (2019) 23(23):10195–203. doi: 10.26355/eurrev_201912_19654 31841172

[174] Zhang Y Guo H Ma L Zhu J Guo A He Y. [Study on adsorption of microRNA-124 by long chain non-coding RNA MALAT1 regulates osteogenic differentiation of mesenchymal stem cells]. Zhongguo Xiu Fu Chong Jian Wai Ke Za Zhi (2020) 34(2):240–5. doi: 10.7507/1002-1892.201906025 PMC8171964 32030958

[175] Fan L Yang K Yu R Hui H Wu W. circ-Iqsec1 induces bone marrow-derived mes-enchymal stem cell (BMSC) osteogenic differentiation through the miR-187-3p/Satb2 signa-ling pathway. Arthritis Res Ther (2022) 24(1):273. doi: 10.1186/s13075-022-02964-x 36517907 PMC9749292

[176] Chen X Ouyang Z Shen Y Liu B Zhang Q Wan L. CircRNA_28313/miR-195a/CSF1 axis modulates osteoclast differentiation to affect OVX-induced bone absorption in mice. RNA Biol (2019) 16(9):1249–62. doi: 10.1080/15476286.2019.1624470 PMC6693548 31204558

[177] Liu S Wang C Bai J Li X Yuan J Shi Z. Involvement of circRNA_0007059 in the regulation of postmenopausal osteoporosis by promoting the microRNA-378/BMP-2 axis. Cell Biol Int (2021) 45(2):447–55. doi: 10.1002/cbin.11502 33200464

